# Retinoic Acid Induces Embryonic Stem Cells (ESCs) Transition to 2 Cell-Like State Through a Coordinated Expression of *Dux* and *Duxbl1*

**DOI:** 10.3389/fcell.2019.00385

**Published:** 2020-01-17

**Authors:** Daniela Tagliaferri, Pellegrino Mazzone, Teresa M. R. Noviello, Martina Addeo, Tiziana Angrisano, Luigi Del Vecchio, Feliciano Visconte, Vitalba Ruggieri, Sabino Russi, Antonella Caivano, Irene Cantone, Mario De Felice, Michele Ceccarelli, Luigi Cerulo, Geppino Falco

**Affiliations:** ^1^Biogem Scarl, Istituto di Ricerche Genetiche “Gaetano Salvatore,” Ariano Irpino, Italy; ^2^Department of Science and Technology, University of Sannio, Benevento, Italy; ^3^Department of Biology, University of Naples Federico II, Naples, Italy; ^4^Department of Molecular Medicine and Medical Biotechnologies, University of Naples Federico II, Naples, Italy; ^5^CEINGE Biotecnologie Avanzate s.c.ar.l., Naples, Italy; ^6^IRCCS-CROB, Referral Cancer Center of Basilicata, Rionero in Vulture, Italy; ^7^Institute of Experimental Endocrinology and Oncology (IEOS), CNR, Naples, Italy

**Keywords:** retinoic acid, metastate, ESCs, 2-cell like, pluripotency

## Abstract

Embryonic stem cells (ESCs) are derived from inner cell mass (ICM) of the blastocyst. In serum/LIF culture condition, they show variable expression of pluripotency genes that mark cell fluctuation between pluripotency and differentiation metastate. The ESCs subpopulation marked by zygotic genome activation gene (ZGA) signature, including *Zscan4*, retains a wider differentiation potency than epiblast-derived ESCs. We have recently shown that retinoic acid (RA) significantly enhances Zscan4 cell population. However, it remains unexplored how RA initiates the ESCs to 2-cell like reprogramming. Here we found that RA is decisive for ESCs to 2C-like cell transition, and reconstructed the gene network surrounding *Zscan4*. We revealed that RA regulates 2C-like population co-activating *Dux* and *Duxbl1*. We provided novel evidence that RA dependent ESCs to 2C-like cell transition is regulated by *Dux*, and antagonized by *Duxbl1*. Our suggested mechanism could shed light on the role of RA on ESC reprogramming.

## Introduction

Embryonic stem cells (ESCs) are derived from the inner cell mass (ICM) of the blastocyst. When cultured in appropriate conditions, they retain their pluripotency, with the ability to differentiate into nearly all embryo cell types (Toyooka et al., 2008; Loh and Lim, 2011). *In vivo* epiblast pluripotency is a transitory state that is maintained *in vitro* through multiple metastable states that fluctuate between self-renewal and differentiation balance, and display a heterogeneous differentiation potential (Ohtsuka et al., 2012). One of these populations marked by *Zscan4* expression retains wider potency capacity, and it is marked by similar expression of 2-cell stage embryo signature, in particular the activation of MERV-L (murine endogenous retrovirus-like) endogenous retrovirus and the expression of *Zscan4* associated gene family among them Prame, Thoc, and Tcstv (Falco et al., 2007; Zalzman et al., 2010; Macfarlan et al., 2012; Cerulo et al., 2014; Eckersley-Maslin et al., 2016). Also, *Zscan4* expressing cells show reprogramming potential and epigenetic hallmark of the early embryonic preimplantation stage (Macfarlan et al., 2012). Under standard ESCs culture conditions, about 3-5% of the whole ESCs population expresses *Zscan4* (Zscan4^+^) which has been shown to mark the ESCs to the so-called “2C-like” transition intermediates (Rodriguez-Terrones et al., 2018).

RA, especially all-trans retinoic acid (ATRA) is the derived form of vitamin A (VitA), and it is involved in a variety of biological functions including embryogenesis, cell differentiation, and apoptosis (Kanungo, 2017). Interestingly, RA enhances Zscan4^+^ up to about 20% of the whole ESCs population (Sharova et al., 2016; Tagliaferri et al., 2016). These effects can be observed in ESCs cultured in RA for long-term, whereby Zscan4^+^ cells emerge within undifferentiated canonical colonies. Recently, we have shown that *Zscan4* RA-dependent activation led to the transition of ESCs to 2C-like state supported by 2-cell stage expression signature, DNA hypo-methylation and global increase of H3K27 acetylation levels (Napolitano et al., 2019).

Although it has been shown that the activation of 2C-like reprogramming is directly regulated through the transcription factor *Dux* and by its positive regulators, *Dppa2* and *Dppa4* (Eckersley-Maslin et al., 2018; De Iaco et al., 2019) the pivotal molecular driver regulation needs further investigation.

RA dependent induction of 2C-like state represents a suitable *in vitro* system to characterize the molecular mechanism orchestrating embryonic-like genome activation and the maintenance of pluripotency.

Here, we demonstrated that RA is necessary for ESCs to 2C-like transition, and reconstructed the gene network underlying 2C-like cell activation by employing reverse engineering *in silico* analysis. We found that RA induction of 2C-like is accompanied by the co-expression of two members of Dux family transcription factors, *Dux* and *Duxbl1*. We investigated the role of these two proteins revealing that such transition requires *Dux* and that Duxbl1 contrasts it. Supporting this, overexpression, chromatin immunoprecipitation (ChIP) analyses and transcription activation assays, revealed that *Dux* and *Duxbl1* coordinate regulation of 2C-like cells through a competitive promoter binding activity.

## Materials and Methods

### Cell Culture

E14Tg2a.4 ES cells, derived from strain 129P2/OlaHsd were purchased from ATCC company and were cultured for two passages on gelatin-coated feeder-free plates and subsequently maintained in gelatin-coated six-well plates in complete ES medium: GMEM (Glasgow Minimum Essential Medium, Gibco), 15% FBS (HyClone), 1,000 U/ml leukemia inhibitory factor (LIF) (Millipore), 1.0 mM sodium pyruvate (Invitrogen), 0.1 mM non-essential amino acids (Invitrogen), 2.0 mM L-glutamine (Invitrogen), 0.1 mM β-mercaptoethanol, and 500 U ml-1 penicillin/streptomycin (Invitrogen). ESCs were incubated at 37°C in 5% CO_2_; medium was changed daily, and cells were split every 2 to 3 days routinely. ESCs were plated in N2B27 (VitA) or N2B27 without retinoids (VitA^minus^) or in the medium supplemented with 1.5 μM all-trans RA(VitA^Plus^), 50 μM citral (all from Sigma-Aldrich) and 5.0 μM BMS493 (Tocris Bioscience). The culture of ESCs with RA was also performed in the presence of 2 μg/ml protein synthesis inhibitor Cycloheximide (Sigma-Aldrich). ESCs were cultured for two passages on gelatin-coated feeder-free in 2i medium, a serum-free N2B27 medium supplemented with MEK inhibitor PD0325901 (0.5 μM) and GSK3 inhibitor CHIR99021 (3 μM) (both from Stemgent), and 1,000 U/ml LIF (Millipore). All experiments were performed at least three times. Dux-ko ES cells were a kind gift of dr. De Iaco and were cultured in 2i medium as described above. HEK293T cells were purchased from ATCC company and were cultured in Dulbecco's modified Eagle's medium (DMEM) supplemented with 10% FBS (Sigma-Aldrich) and 1% Pen/Strep (Sigma-Aldrich).

### Plasmids

To generate pZscan4c-pcDNA3.1/CT-GFP-TOPO constructs, a putative *Zscan4c* promoter (pZscan4) corresponding to −2,400, −480, and −288 bp, respectively, from the *Zscan4c* Transcription Start Site (TSS) was amplified from BAC RP23-63I1. The *pZscan4* was amplified using primers: 5′-TTCTTAATCTGTGGTCGTCCA-3′; 5′-TGTGGTGACAATGGTGTGAA-3′; 5′-GCCAATCTTGGAATTCCTCTTC-3′; 5′-TTGCTTGTATTTGATTCCCC-3′. Dux-HA was a kindle gift of De Iaco. Duxbl1-V5 was obtained cloning Duxbl1 coding sequence into pcDNA3-V5 His (Invitrogen). Duxbl1_CTD-Flag was obtained using the In-Fusion cloning strategy (Takara).

### Site-Directed Mutagenesis

To generate mutant plasmid for *Duxbl1* binding site, QuikChange Lightning Site-Directed Mutagenesis Kit was used according to the manufactures instruction. Briefly, ML_480 pZscan4-pcDNA3.1/CT-GFP-TOPO with WT-Duxbl1 binding motif was amplified using two synthetic oligonucleotide primers, carrying the mutation for Duxbl1 motif (fw 5′-GAAAGACATTTTTTCCTGCTGAGCCGGTCACATAAGGAATCCTAACTCAGCTCTAGTTTTGCATCTC-3′; RV 5′- GAGATGCAAAACTAGAGCTGAGTTAGGATTCCTTATGTGACCGGCTCAGCAGGAAAAAATGTCTTTC-3′). Sequence analysis confirmed the presence of the mutation.

### Flow Cytometry and Sorting of pZscan4-GFP ESCs

pZscan4-GFP cells were fed at least 2 h before harvesting by Trypsin (Gibco) and resuspended in complete ES medium containing 25 mM HEPES buffer. The cells were then FAC-sorted according to the fluorescent intensity of GFP into complete ES medium containing HEPES. Data are presented as mean % ESZscan4_GFP cells ± SEM of three independent experiments with statistical analysis performed using Student's *t*-test.

### Generation of E14tg2a.4pcDNA3_pZScan4_LNGFR Stable Cell Line

For the construction of the plasmid pcDNA3_pZscan4_LNGFR, Zscan4 promoter was amplified by PCR from pZScan4-GFP vector and inserted into KpnI/EcoRV sites of pcDNA3 vector (Invitrogen). Subsequently, LNGFR fragment (874 bp) was amplified by PCR from pPRIME-CMV-LNGFR and inserted into EcoRV site of pcDNA3_pZScan4 vector. The construct was verified by sequencing. To generate the stably transfected ES cell line pcDNA3_pZScan4_LNGFR was linearized with KpnI and transfected into wild-type E14tg2a.4. 48h after transfection the cells were split and positive clones selected for Neo resistance. After 1 week of G418 (Gibco) treatment, NeoR clones were picked and propagated.

### E14tg2a.4pcDNA3_pZScan4_LNGFR Culture and Magnetic Separation

The stably transfected ESCs were cultured for 3 days on gelatin-coated dishes in ES complete medium. The cells were then trypsinized and plated on gelatin-coated dishes in N2B27-VitA medium: KnockOut DMEM high glucose (Gibco) supplemented with L-Glutamine 2 mM (Gibco), Penicillin/Streptomycin 100 U-μg/ml (Gibco), B27-VitA Supplement 1x (Gibco), N2 Supplement 1x (Gibco), 2(β)Mercaptoethanol 0.1 mM (Gibco), LIF 1,000 U/ml (Millipore), G418 137,5 μg/ml, with or without 1.5 μM RA for 72 h. For magnetic labeling, single-cell suspensions were centrifuged, resuspended in PBS supplemented with 5 mM EDTA and 0.5% BSA and incubated with MACSelect (TM) LNGFR MicroBeads for 15 min on ice. Magnetically labeled cells were isolated over the AutoMACS Pro Separator (MiltenyiBiotec) with “posseld2” program according to the manufacturer's protocol. For purity assessment, aliquots of original cell population (magnetically labeled cells before separation), eluted positive (enriched target cells) and negative (untargeted cells collected in the flow-through fraction) cell populations were fluorescently stained with MACSelect Control FITC Antibody (MiltenyiBiotec) that specifically stains MACSelectMicroBead-labeled cells and analyzed by Navios Flow Cytometer (Beckman Coulter).

### RNA Extraction and qPCR Analysis

For qPCR analysis of sorted cells, total RNAs were collected immediately after sorting by TRIzol (Invitrogen) according to the manufacture's instructions. One microgram of total RNA was reverse-transcribed by Quantitec reverse transcription kit (Qiagen) according to the manufacturer's instructions. qPCR analyses were performed using 10 ng cDNA per well in duplicate with the SYBR green master mix (Applied Biosystems) as previously described (Falco et al., 2006; Vivo et al., 2017). Reactions were run on QuantStudio 7 Flex system and 7900 realtime Pcr system (Applied Biosystems). Fold induction was calculated using ΔΔCt method while the normalization was performed using *Gapdh or* the mean of three housekeeping genes: *Gapdh, Actin and 18S* (16). The gene-specific primers are available in Table S4.

### RARE Motif Analysis

The promoter sequences of Zscan4 were retrieved from assembly mm10 of the mouse genome. The known consensus sequences of RARE motifs (Bastien and Rochette-Egly, 2004) were converted into position weight matrixes using upac2meme conversion tool provided by MEME suite. Positive controls known in the literature as primary gene responsive to RA were adopted to estimate a suitable q-value threshold (Bastien and Rochette-Egly, 2004; Cunningham and Duester, 2015).

### Global Regulatory Network Reconstruction

We selected a collection of 754 gene expression profiles in the context of mouse ESC where 181 ESC related transcription factors were either up-regulated or repressed (GEO Accession Numbers: GSE31374, GSE14559, GSE26520). The expression profiles were normalized with a quantile normalization approach, and each probe was associated with the corresponding gene symbol based on annotation information included of the chip NIA Mouse 44K Microarray v3.0 (Whole Genome 60-mer Oligo). We averaged all significant redundant probes for the same gene. Both control and not annotated probes were removed from the analysis obtaining an expression matrix of 24,988 genes in 754 conditions. The regulatory network was learned with a three steps procedure similar to ARACNE (Basso et al., 2005) consisting of: (i) computation of mutual information between 1,852 Transcription Factors (TFs) obtained both from a manually curated collection based on Gene Ontology and from AnimalTFDB database (Zhang et al., 2012) and 24,988 gene expression profiles in 754 conditions to determine statistical dependence between transcription factors and target genes (Basso et al., 2005); (ii) Data processing inequality to filter out indirect relationships (Sales and Romualdi, 2011); and (iii) Permutation test to keep only statistically significant relationships. In particular, for each link, we obtained the null distribution by recomputing 1,000 times the mutual information of the link with a randomly permuted expression profile of one of the two genes. We retained only links with and FDR ≤ 0.01.

### Master Regulator Analysis (MRA)

MRA is widely adopted to identify Master Regulator (MR) transcription factors acting in a particular context of interest (Lefebvre et al., 2010). The enrichment, evaluated using a statistical test such as Fisher's exact test or GSEA (Subramanian et al., 2005), has the objective to place the signature genes within a regulatory context in order to identify the master regulators responsible for coordinating their activity, thus highlighting the regulatory apparatus driving the functional phenotype of interest.

We computed the enrichment of each TF with GSEA using the *fgseaMultilevel* function of fgsea R package (Sergushichev, 2016). We computed the statistical significance of the enrichment by performing 10,000 permutations, followed by multiple hypothesis testing with Benjamini Hochberg adjustment, obtaining a set of 266 candidates (FDR ≤ 0.01) (Table S2).

### *Zscan4* Promoter Analysis

To verify if a candidate MR directly regulate the expression of Zscan4, promoter analysis was performed using the Biostrings (Pagès et al., 2019) package function matchPWM. The statistical significance of each predicted PWM match was performed using the TFMPvalue package TFMsc2pv function (Touzet and Varré, 2007). The Zscan4 promoter sequence was retrieved from assembly mm10 of the mouse genome and scanned against a list of available PWM matrices related to MRs and obtained from MEME (JASPAR 2016 CORE and Vertebrates, CIS-BP and Mouse Uniprobe PWM collections). We obtained a list of putative motifs that bind the promoter region of Zscan4 (normalized binding score ≥ 0.7 and *p*-value ≤ 0.0001, Table S3).

### Chromatin Immunoprecipitation Assay

Approximately 1.5 × 10^7^ ESCs were grown at 70-80% confluency. The cells were cross-linked by adding fresh 0.75% formaldehyde solution to the ES media for 10 min at room temperature, and they were treated with 125 mM glycine for 5 min. Cells were lysed and sonicated to solubilize chromatin and shear the cross-linked DNA. Sonications were performed at power 10 for 6 × 30 second pulses (30 s pause between pulses) at 4°C. Fifty microliters of each sonicated sample was removed and used to quantify the DNA concentration and as a control in the PCR. Fifty micrograms of DNA were incubated overnight on a rotating platform at 4°C with 20 μl of Protein A/G beads and with 10 μg of specific antibody. Immunoprecipitated chromatin was eluted, treated with 20 mg/ml Proteinase-K and was purified by phenol:chloroform:isoamyl alcohol extraction. DNA levels are quantitatively measured by qPCR. The primers used for qPCR to amplify the *Duxbl1* were 5′-TGGAATTCCTCTTCAGTGTGG-3′ and 5′-ATTCCCCCTTTTGGCATTAT-3′ resulting in a product size of 217 bp, and primers used for the negative control were 5′-ACCAACTCCAGCTAAGGGGA-3′ and 5′-GGCAGAGGTGTGTGCATACT-3′. The antibodies used for chromatin immunoprecipitation are: anti-FLAG (Sigma Aldrich), anti H3 acetyl (Upstate).

### Western Blot Analysis

Total protein was extracted with cell extraction buffer using the following formulation: 100 mMTris pH 7.4, 2 mM Na3VO4, 100 mM NaCl, 1% NP40, 1 mM EDTA, 1 mMNaF, 0.5% deoxycholate, 20 mM Na4P2O7, 1 mM PMSF, and 1X Protease Inhibitor Cocktail (Sigma) as previously described (Di Martino et al., 2016; Fontana et al., 2018). Protein concentrations were determined using the Bio-Rad protein assay kit according to the manufacturer's instructions. Twenty micrograms of protein lysate were separated on SDS-PAGE and transferred onto a nitrocellulose membrane. The following primary antibodies were used: anti-FLAG (1:1,000, Sigma Aldrich), anti-GAPDH (1:1,000, Genetex) anti Zscan4 (1:5,000, Millipore) anti-Actin (1:10,000, Sigma Aldrich) (Figure S1). The membranes were incubated with antibodies to specific proteins followed by incubation with HRP-conjugated anti-rabbit IgG or anti-mouse IgG (1:2,500; Santa Cruz Biotechnology).

### Luciferase Assay

The 1,330 bp*Zscan4c* promoter was inserted into a pGL3 vector (Clontech). An expression plasmid (pCDNA3.1/FLAG) containing the full length of mouse *Duxbl1* sequence was constructed. Dux-HA was a kind gift of dr. De Iaco. To assess *Zscan4* activation state, HEK293T cells were transfected with Dux-HA alone and with increasing concentration of Duxbl1-FLAG together with pGL3-Basic vector (Clontech) in 60 mm plates. The RSV-β-galactosidase plasmid was added to transfection mixtures to normalize the luciferase values for the efficiency of transfection. Twenty-four hours after transfection, luciferase activity was determined using the Luciferase Assay System (Promega, Madison, WI, USA).

## Results

### Retinoic Acid (RA) Signaling Is Required for Zscan4 Metastate Activation

**Figure 1:**
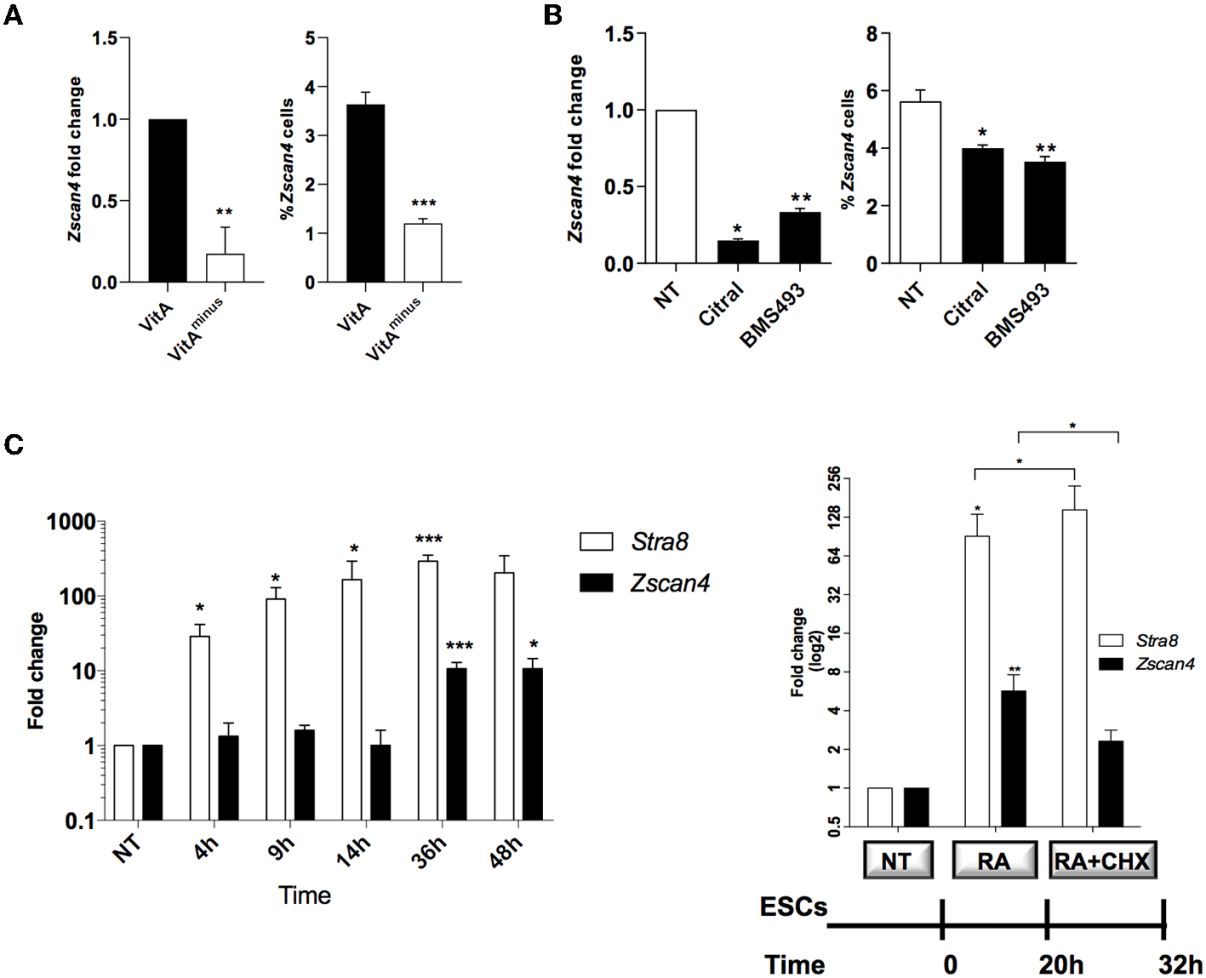
Role of retinoids on Zscan4 cell population induction. **(A)** ESCs, cultured for 72 h in VitA and VitA^*minus*^ condition, were analyzed by qPCR and cytofluorimetry. The *Zscan4* expression levels were normalized to *Gapdh* expression and fold induction compared to VitA (left); the percentage of Zscan4^+^ cells was evaluated by flow cytometry analyses (right). **(B)** *Zscan4* expression levels were assessed in ESCs cultured for 72 h with Citral or with BMS493 by qPCR, normalized to *Gapdh* expression and compared to NT (left); percentage of Zscan4^+^ cells was evaluated as in **(A)** (right). **(C)** ESCs, cultured in the presence of RA for 4, 9, 14, 36, and 48 h, were analyzed for *Stra8* and *Zscan4* expression (left). ESCs were grown in the presence of 1.5 μM RA for 32 h and in the absence/presence of 2 μg/ml cycloheximide (CHX) for 12 h (right). *Stra8* and *Zscan4* expression levels were assessed by qPCR and expressed as fold change respect to NT condition. The normalization was performed using the mean of three different housekeeping genes (*Gapdh, Actin, 18S*). The average and SEM of all the experiments were performed on the duplicate samples from three independent biological experiments and are shown: *p < 0.05, **p < 0.01, ***p < 0.001, in a Student's *t*-test.

We have previously shown that retinoids enhance induction of the so-called 2C-like cells marked by 2-cell embryo stage signature, in particular by *Zscan4* (Figure S1, Tagliaferri et al., 2016). To assess how retinoids regulate the transition of ESCs to 2C-like cell population, we grew ESCs in culture medium with or without vitamin A and measured *Zscan4* expression level and the percentage of Zscan4^+^ cells. ESCs cultured for 72 h in retinoids-free N2B27 medium (hereafter VitA^*minus*^) showed a decrease of about 80% of *Zscan4* expression compared to N2B27 (which contains traces of retinoids, hereafter VitA) as measured by quantitative PCR (qPCR) (Figure 1A, left). Coherently, cytofluorimetric analyses on ESCs transgenic line in which the expression of *Zscan4* can be reported by GFP (ESC^*pZscan*4_*Em*^) (Tagliaferri et al., 2016) showed that the percentage of Zscan4^+^ cells was reduced from about 4% to about 1% from VitA to VitA^minus^ cell culture media (Figure 1A, right). Concurrently with *Zscan4* downregulation, in VitA^*minus*^ condition, also *Zscan4* associated genes, including *Eif1a, Gm12794, Gm4340*, and *Tcstv1* (Figure S2), were significantly repressed.

We next cultured ESCs with or without citral, an inhibitor of aldehyde dehydrogenase that blocks the conversion of retinol to RA. Moreover, we treated ESCs with the RA-receptor (RAR) inhibitor, the BMS493 molecule. In the presence of either citral or BMS493, *Zscan4* expression was significantly diminished (Figure 1B, left) as well as the fraction of Zscan4^+^ (Figure 1B, right), compared to not treated sample (NT). Collectively our results indicate that both RA biosynthesis and functional RAR signaling enhance the transition of ESCs to Zscan4^+^ cell population.

RA signaling acts through the regulation of primary and secondary-response genes. Primary genes are induced within about few hours upon RA stimulus and do not rely on *de novo* protein synthesis, while secondary genes are transcribed at later times only once their regulators have been synthesized (Balmer and Blomhoff, 2002; Tullai et al., 2007). To investigate the mechanism of action of RA we indeed performed time-course experiments in which ESCs were treated with RA for 4, 9, 14, 36, and 48 h. The qPCR analyses showed a significant increase of *Zscan4* levels starting at 36 h, consistently with a secondary response (Figure 1C, left). Conversely, expression levels of a well-known RA primary response gene, *Stra8*, were significantly higher than the control levels (about 40-folds) as early as 4 h post-RA treatment (Figure 1C). These observations suggest that *de novo* synthesized regulator proteins are required to induce *Zscan4*. To address this hypothesis, we cultured ESCs in the presence of RA for 20 h and then incubated with the protein synthesis inhibitor cycloheximide (CHX) or control medium for additional 12 h (Figure 1C, right). The addition of CHX to the culturing medium significantly impaired *Zscan4* transcription of about 50% (Figure 1, right) while, as expected, *Stra8* was not affected.

### RA Induces the Transition of ESCs to 2C-Like Cell Through *Zscan4* Intermediates

**Figure 2:**
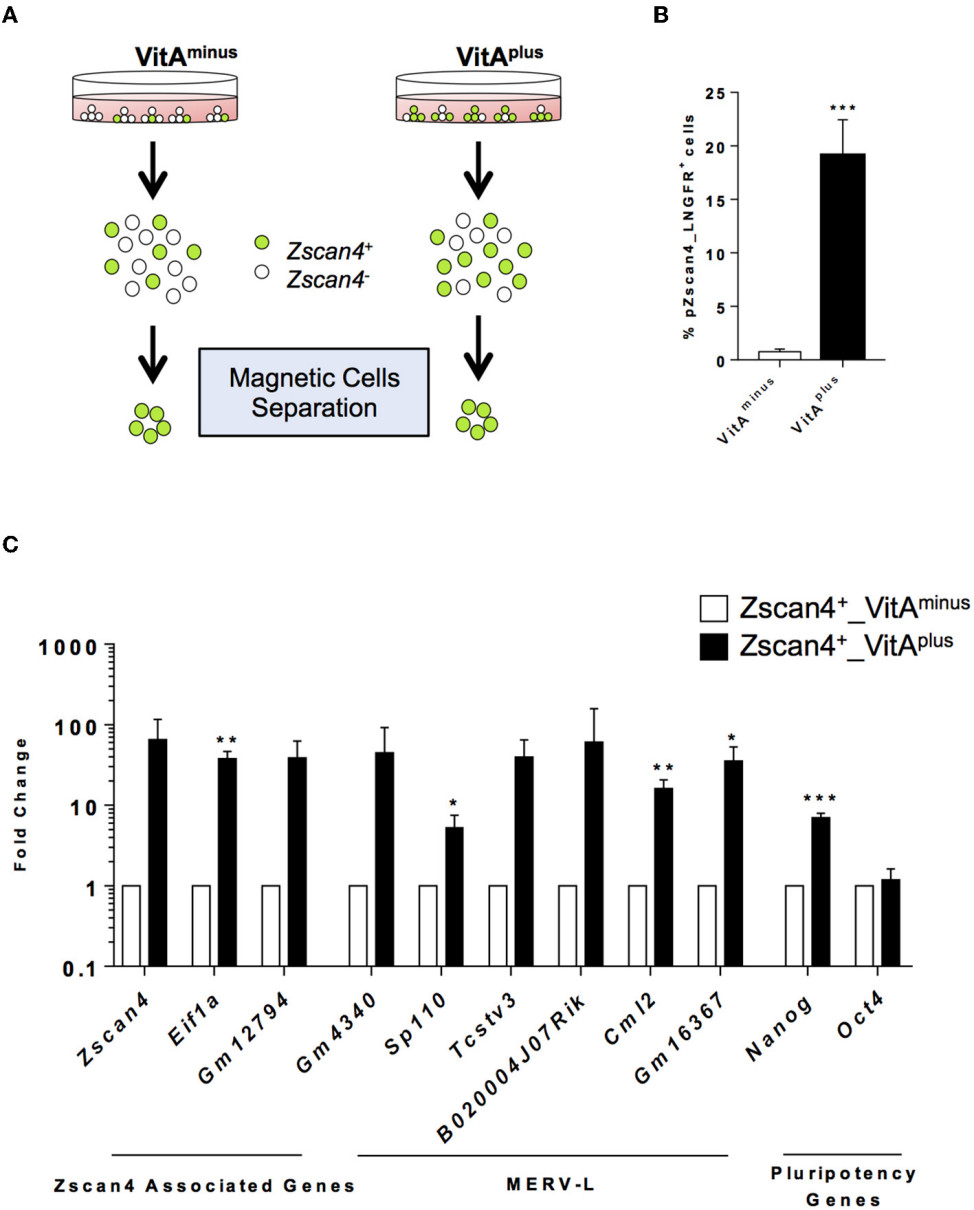
RA effects on ESCs to 2C-like transition. **(A)** Schematic illustration of the system used for Zscan4^+^ cells magnetic separation. Zscan4 promoter drives the expression of the extracellular portion of the human cell surface receptor LNGFR. **(B)** The modified ES^Zscan4_LNGFR^ cells were cultured in VitA^minus^ and VitA^plus^ for 72 h and incubated with a LNGFR magnetically labeled antibody. Positive fractions were collected through autoMacs Separator. The histogram describes the percentage of separated cells in the different culture conditions. **(C)** Analysis of genes upregulated during the ZGA. The gene expression levels were assessed by qPCR as fold change to VitA^minus^ condition and the normalization was performed using the mean of *Gapdh, Actin* and *18S*. Statistical significance was calculated by Student's t-test (*p < 0.05, **p < 0.01, ***p < 0.001).

The exit from the ESCs toward the 2-cell-like state is a multistep process by which cells encompass several intermediate states, determined by the levels of *Zscan4* expression, each characterized by a known and specific molecular signature (Rodriguez-Terrones et al., 2018). We explored the hypothesis that RA signaling could induce Zscan4 intermediate transitions. Since *Zscan4* expressing cells are scarcely abundant in the medium not supplemented with RA (Figure 1A), we designed a system to efficiently and quickly collect Zscan4 subpopulation. In particular, we generated a modified ESCs line harboring the extracellular portion of the human low-affinity nerve growth factor receptor gene (*LNGFR*) under the control of the *Zscan4* promoter (Napolitano et al., 2019). This strategy allowed us to efficiently separate and collect Zscan4^+^ cells (Figure S3), overcoming long rounds of FAC-sorting. Next, we cultured ESCs^*Zscan*4−*LNGFR*^ for 72 h either in VitA^minus^ or in medium supplemented with Retinoic Acid (hereafter VitA^*plus*^). Subsequently, cells were isolated using a magnetically labeled anti-LNGFR antibody (Figure 2A). As expected the percentage of Zscan4^+^ cells in the absence of RA is extremely low (less than 1%) compared to the number of positive cells upon RA treatment (about 20%) as shown in Figure 2B. We next compared *Zscan4* levels in LNGFR positive cells derived from the different culture conditions by qPCR (Figure 2C).

Interestingly, *Zscan4* expression levels were higher in positive cells isolated from VitA^plus^ than positive cells from VitA^minus^ (Figure 2C). To investigate whether these two populations (“Zscan4^high^” and “Zscan4^low^”) corresponded to the intermediate states arising during ESCs to 2C-like transition, we also analyzed the expression of MERV-L genes that are transcriptionally activated during ZGA (Macfarlan et al., 2012). Expression analysis revealed that MERV-L genes were either significantly upregulated or exclusively expressed in Zscan4^high^ compared to Zscan4^low^ cells (Figure 2C). These data suggest that RA induces 2C-like state by acting on the transition of Zscan4^low^ (less than 1% prior to RA activation) to Zscan4^high^ intermediates (Figure 2B, Supplementary Video).

### Functional Genomic Analyses Reveal *Zscan4* Transcription Regulators

**Figure 3:**
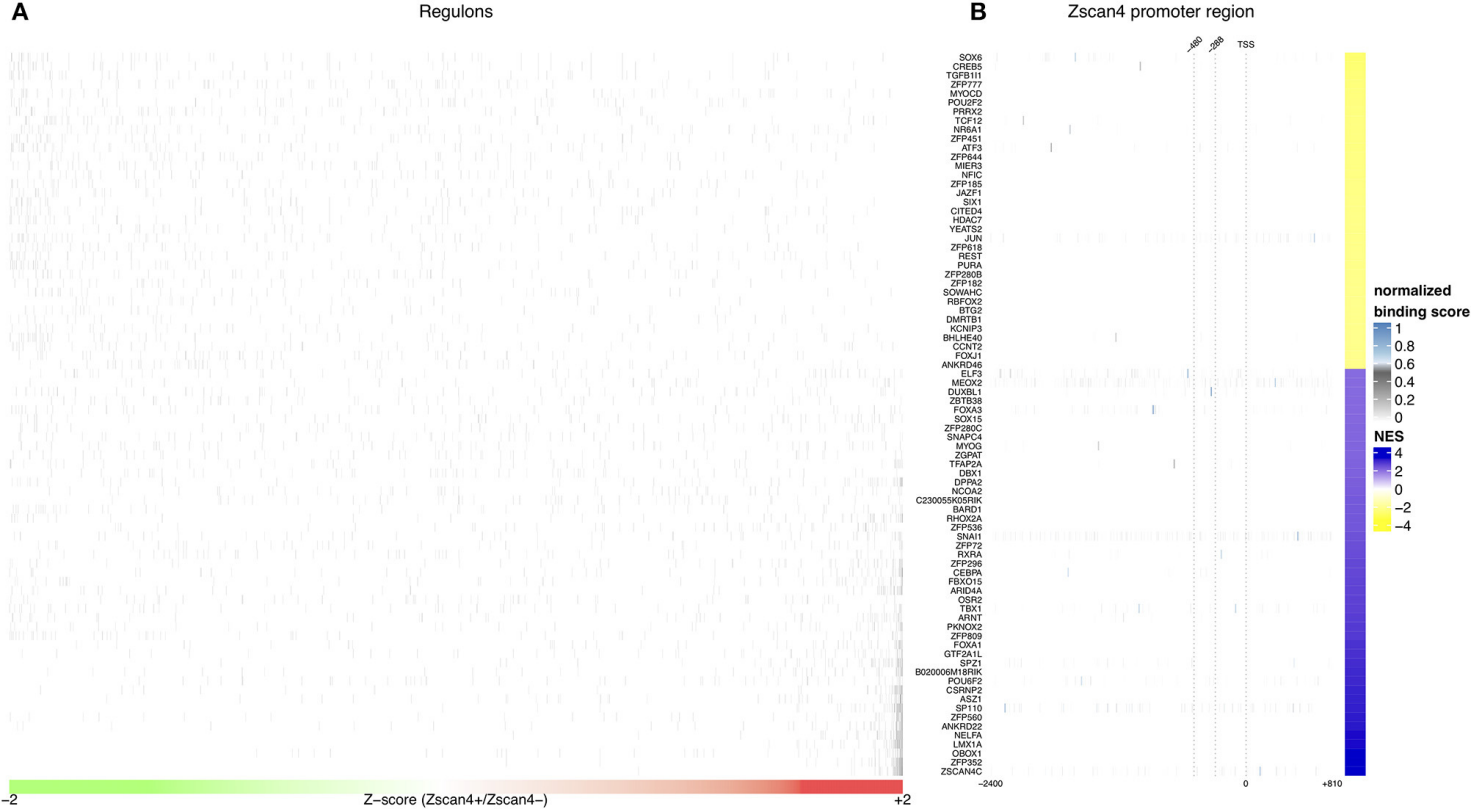
*In silico* analysis of *Zscan4* regulatory network. **(A)** Blue and Yellow colors indicate positive vs negative regulation of Master Regulators (MRs). Overall were found 80 significant master regulators. The figure shows, for each significant master regulator, the distribution of regulons respect to RA-Zscan4^+^ vs. RA-Zscan4^−^ metastate phenotype. **(B)** The figure shows significant binding activity in the promoter region of *Zscan4c*.

Given the crucial role of RA on activation of Zscan4^low^ state, we searched for intermediate regulators of *Zscan4* transcription that could shed light on the molecular networks underlying RA-dependent *Zscan4* induction. To verify whether Zscan4 is directly regulated by retinoids, we analyzed the promoter region of *Zscan4* (2,400 bp upstream the TSS) to scan the presence of retinoic acid response elements (RARE motifs). To this aim, we adopted the FIMO (“Find Individual Motif Occurrences”) tool that computes a log-likelihood ratio score and a q-value for each position in a given sequence (Grant et al., 2011). In line with our previous findings, *in-silico* analysis of a 2,400 bp region upstream the Zscan4 promoter by FIMO did not result in any canonical RAR elements (RARE) identification (Table S1, *q*-value < 0.1) consistently with our previous findings that *Zscan4* is a RA secondary-response gene (Figure 1C). To identify putative Zscan4 direct and indirect regulators, we reconstructed a global regulatory network from a wide collection of ESCs specific gene expression profiles using a consolidated reverse engineering approach (Figure S4) (Liu, 2015). We adopted a genome-wide reverse engineering approach that integrates both sequence (putative binding motif) and functional expression data. We used a set of 754 ESC-specific expression profiles to reconstruct a global regulatory network surrounding *Zscan4*. This network consists of 476.654 interactions among 1.852 Transcriptional Factors (TFs) and 24.988 genes (Figure 3A, Figure S4).

To better underlying the transcriptional mechanism acting for the maintenance of Zscan4^+^ metastate under RA effect, we adopted the Master Regulator Analysis (MRA) algorithm to compute the statistical significance of the overlap between the regulatory targets of each TF and the Zscan4 signature represented by the list of differentially expressed genes enriched in RA-Zscan4^+^ compared to RA-Zscan4^−^ (the dataset was previously published, Tagliaferri et al., 2016, GEO accession number: GSE75977). This analysis allowed us to identify 80 significantly enriched [*p*-value ≤ 0.001 and absolute Normalized Enrichment Score (NES) greater than 70th percentile] Master Regulator (MR) candidates to be regulators of the RA-mediated Zscan4^+^ metastate (Table S2). To identify direct regulators, we performed an additional sequence motif binding analysis to excluded MRs with no evident binding signals on *Zscan4* promoter. We identified 4 MRs that exhibited significant promoter binding activity (*p*-value ≤ 0.0001 and normalized binding score ≥ 0.7) (Figure 3B, Table S3): Elf3 (E74 like ETS transcription factor 3), Sox6 (Sry type HMG box 6), Foxa3 (Fork head box A3), and Duxbl1 (Double homeobox B-like 1).

### Retinoids Induce *Zscan4* Expression Mediating Dux Binding Activity

**Figure 4:**
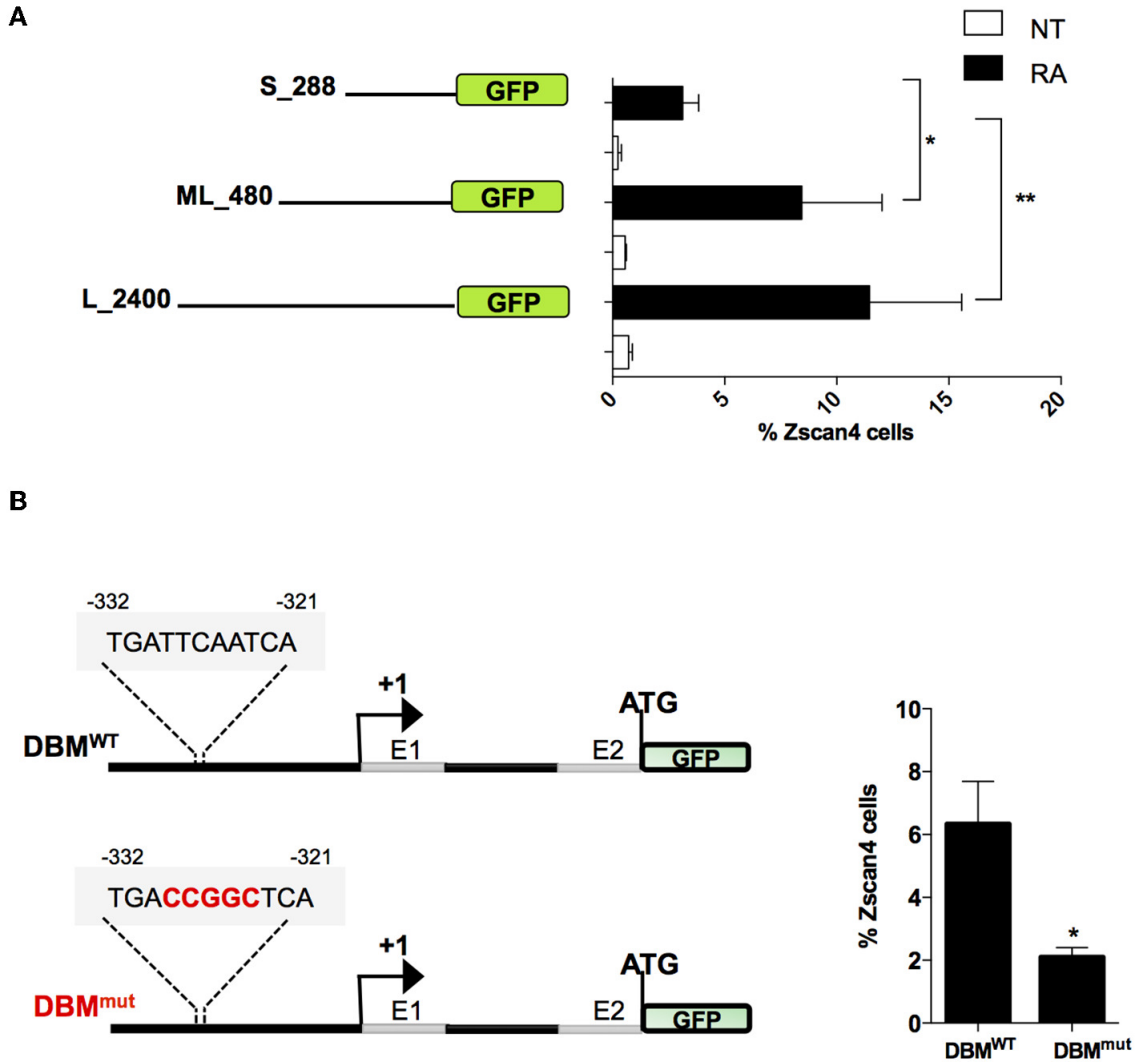
Evaluation of RA responsive DNA element on *Zscan4* promoter. **(A)** Flow cytometry analysis of ESC^Zscan4_GFP^ lines expressing GFP under *Zscan4* promoter regions of: S_288 (short), ML_480 (Mid-length) and L_2400 (Long) starting from Transcription Start Site (TSS). ESC^Zscan4_GFP^ lines were cultured for 5 days in media with or without RA. **(B)** Schematic representation of genetic constructs of ML_480 *Zscan4* promoter that contains either wild type Dux Binding Motif (DBM^WT^) or mutant (DBM^mut^) at about −330 bp from the transcription start site (TSS) (left). ESCs^DBM−WT^ and ESCs^DBM−mut^ were cultured in RA for 5 days, and the percentage of Zscan4^+^ cells were analyzed by cytofluorimetry assay. The average and SEM of three independent biological experiments are shown: *p < 0.05, **p < 0.01 in a Student's *t*-test.

To narrow down the list of MRs we characterized the minimal region of *Zscan4* promoter responsive to RA. In particular, we evaluated the promoter activity of deletion mutants upon RA treatment by mean of a GFP reporter assay. The analyses were conducted on three overlapping *Zscan4* promoter regions cloned upstream GFP: the 2,400 bp (long, L), the 480 bp (mid-length, ML) and the 288 bp (short, S) fragments (Figure 4A). The corresponding genetic constructs were stably transfected in ESCs. Selected clones were grown with or without RA, and GFP expression was analyzed through cytofluorimetric assay.

Interestingly, the fraction of GFP-positive cells harboring the short promoter form poorly responded to RA (Figure 4A). These results allowed us to map the *retinoic acid responsive region for Zscan4 activation* between −288 and −480 bp upstream the start the Transcription Start Site (TSS).

Among the subset of 4 MRs previously identified, Duxbl1 resulted the only one having a significant binding activity in this region. Consistently, canonical Dux Binding Motif (DBM), has been previously mapped on *Zscan4* promoter (Geng et al., 2012; De Iaco et al., 2017; Hendrickson et al., 2017). To determine whether this Dux binding motif was required for RA-dependent *Zscan4* transcriptional activation, we generated a transgenic ESCs line expressing GFP under the control of *Zscan4* promoter harboring mutated (DBM^mut^) or wild type (DBM^WT^) DBM sequence (Figure 4B, left). Following RA treatment, the percentage of fluorescent cells in the two clones was measured by citofluorimetry. The experiment showed that the fraction of Zscan4^+^ was reduced about 70% (from 6 to 2%) comparing DBM^WT^ vs. DBM^mut^ (Figure 4B, right). Overall, our data showed that RA activates *Zscan4* through DBM region.

### Dux and Duxbl1 Directly Bind and Regulate *Zscan4* Expression

**Figure 5:**
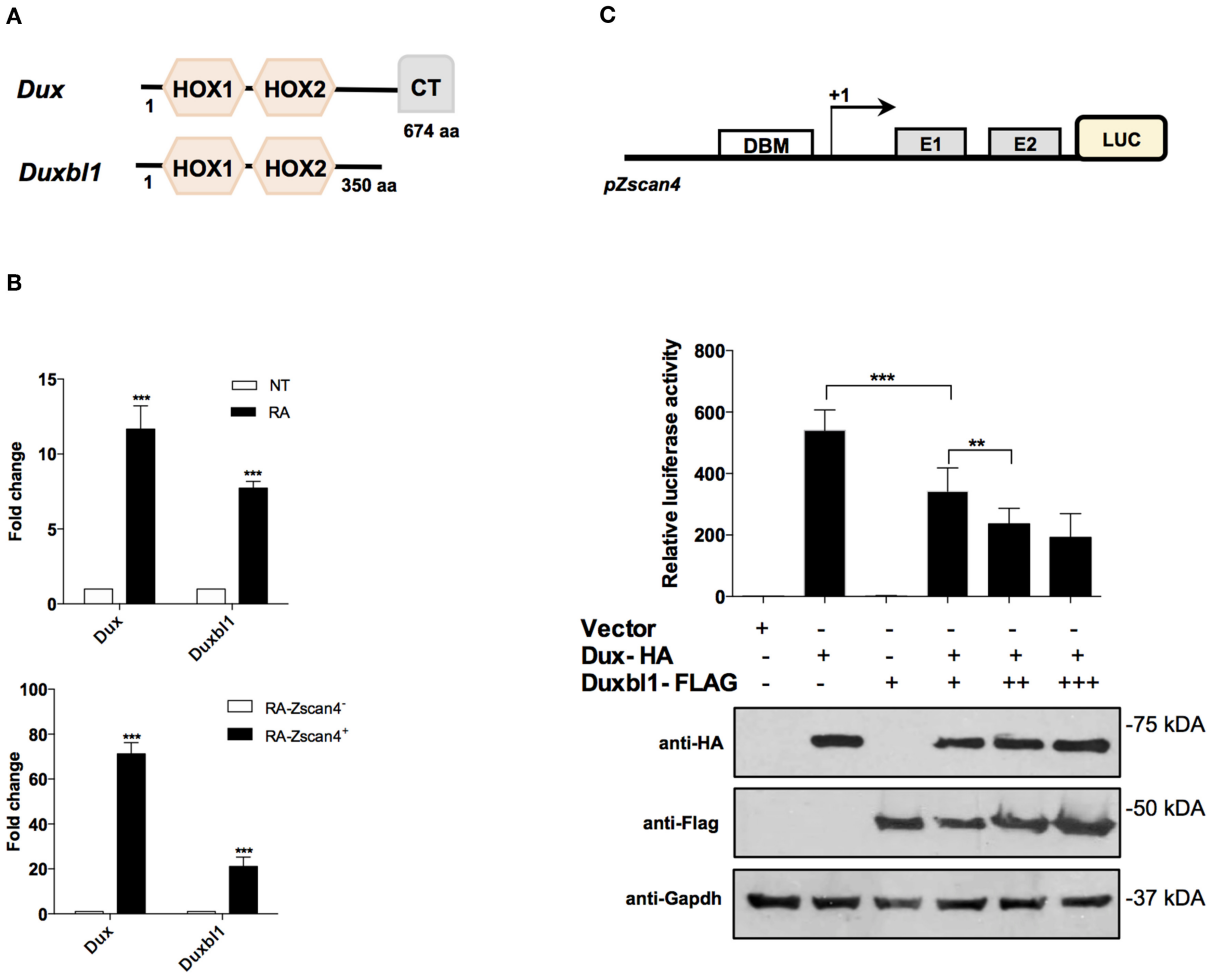
Role of Dux *and* Duxbl1 *on Zscan4* promoter regulation. **(A)** Schematic representation of Dux and Duxbl1 protein domains. **(B)** *Dux* and *Duxbl1* expressions were significantly induced by RA. The normalization was performed using Gapdh as housekeeping gene. (top). ES^Zscan4_Em^ cells grown for 5 days with or without RA and sorted by flow cytometry. *Dux* and *Duxbl1* expressions were robustly enriched in Zscan4^+^ cells (bottom). The normalization was performed using Gapdh as housekeeping gene. **(C)** HEK293T cells with Luciferase-reporter gene under Zscan4 promoter regulation (scheme) were transiently co-transfected with: control Vector, or Dux-HA, Duxbl1-Flag either alone or with Dux-HA. Data shown represent relative luciferase activity normalized against the β-galactosidase activity, in the top panel, while in the bottom panel was reported the western blot assay corresponding to luciferase assay. Dux-HA and Duxbl1-Flag were detected with anti-HA, anti-Flag antibodies while anti-Gapdh was used for proteins normalization. *p < 0.05, ***p < 0.01, ***p < 0.001 in a Student's *t*-test.

The Dux family proteins contain a double DNA binding homeodomain (HOX1 and HOX2) and a variable carboxyl-terminal domain (CT) (Figure 5A). The C-terminal domain tethers the histone acetyl-transferase p300 to chromatin, thereby enhancing the transcriptional activation of target genes, such as *Zscan4* (De Iaco et al., 2017). Comparing the protein structure of Dux and Duxbl1, they differ at the C-terminus as Duxbl1 is devoid of CT region (Figure 5A) that suggest an opposite effect on the regulation of their targets.

We validated the hypothesis that *Dux* and *Duxbl1* are part of *Zscan4* regulatory network under RA action. First, we analyzed whether *Dux* and *Duxbl1* expression in ESCs was induced by RA. Two days after RA-treatment, the qPCR analysis showed that *Dux* levels were increased 12-fold on average while *Duxbl1* increased 8-fold (Figure 5B, top). Second, we assessed whether their expression was preferentially induced in Zscan4^+^ cells. To this aim, we employed a transgenic ESC^Zscan4_GFP^ line to isolate Zscan4^+^ and Zscan4^−^ cell population by mean of FAC-sorting from RA medium. The experiment showed *Dux* is expressed 60 times higher in Zscan4^+^ than Zscan4^−^, while a 20 times increase was observed for *Duxbl1* (Figure 5B, bottom).

Based on these results, we thus explored the hypothesis that under RA stimuli *Dux* and *Duxbl1* have opposing functions in determine *Zscan4* activation. To this aim, we generated a HEK293T reporter line in which the luciferase is under *Zscan4* promoter (Figure 5C). Overexpression of transfected Dux-HA significantly enhanced luciferase activity (Figure 5C), coherently reproducing the Dux capability to activate *Zscan4* expression (Choi et al., 2016). In contrast, Duxbl1-Flag over-expression did not induce luciferase activity. Notably, the effect of Dux on luciferase activity was significantly hampered by co-transfecting increasing amount of Duxbl1 (Figure 5C). In addition, in order to further demonstrate the competition between Dux and Duxbl1 for Zscan4 promoter activation, we generated a mutant of Duxbl1 carrying the Dux C-terminal transactivation domain (CTD). The luciferase assay (Figure S5) shows that Duxbl1 mutant acquires the capability to activate Zscan4 promoter confirming the hypothesisthat Dux and Duxbl1 parallelly binds and co-regulate *Zscan4* with contrasting effects on its expression.

### *Duxbl1* Hampers ESCs to 2C-Like Transition by Directly Counteracting *Zscan4* Activation

**Figure 6:**
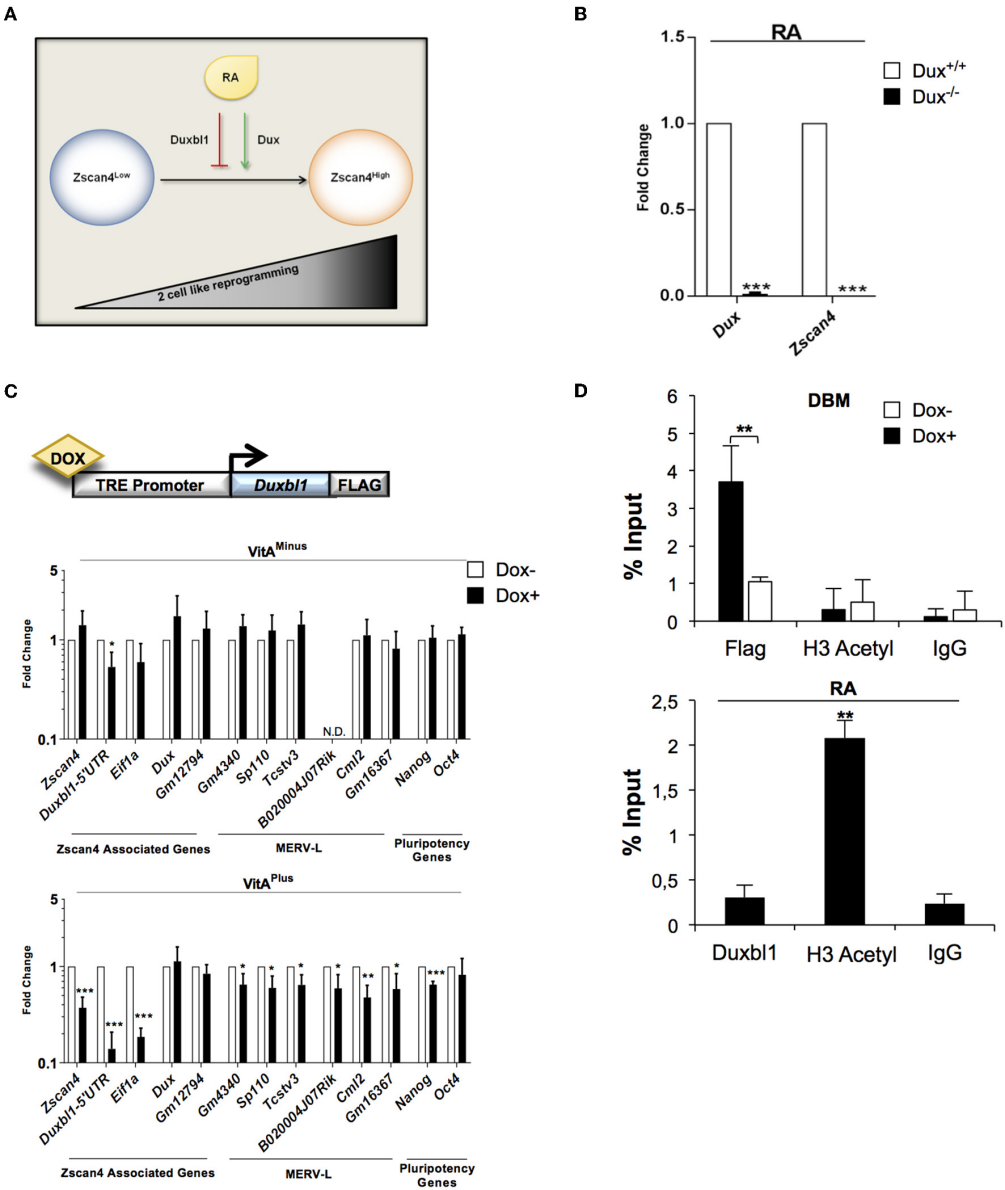
*Dux* and *Duxbl1* effect on RA dependent ESCs to 2C-like transition. **(A)** Model of ESCs to 2C-like transition involving RA dependent Dux and Duxbl1 regulation. **(B)** Dux^−/−^ ESCs were cultured in RA supplemented medium for 4 days. The *Dux* and *Zscan4* expression levels were assessed by qPCR, normalized using *Gapdh* as housekeeping gene and expressed as fold change compared to Dux^+/+^ condition. The average and SEM of all the experiments were performed on three independent biological experiments and are shown: *p < 0.05, **p < 0.01, ***p < 0.001 in a Student's *t-*test. **(C)** Schematic representation of Duxbl1 tetracycline-inducible system (top). ESC^Duxbl1−Flag^ transgenic lines were cultured with or without DOX (1.5 μg/ml for 72 h), in VitA^minus^ and VitA^plus^ conditions. qPCR analysis was performed for Zscan4 associated markers using the mean of three different housekeeping genes (*Gapdh, Actin, 18S*) (bottom). **(D)** In the top, ESC^Duxbl1−Flag^ cells were cultured in presence or not of DOX and chromatin was analyzed by ChIP assay using: anti-Flag, anti-H3Acetyl and IgG antibodies. In the bottom, ESCs treated with RA for 5 days were analyzed through ChIP with: anti-Duxbl1, anti-H3Acetyl and IgG antibodies. Both ChIP assays were analyzed by qPCR for the binding to Zscan4 promoter, expressed as percentage of input.

Our data collectively suggested that *Duxbl1* hampers *Zscan4* expression by competing with *Dux* activity. This prompted us to investigate whether *Dux* and *Duxbl1* could function as agonist and antagonist of ESCs transition to 2C-like cells, respectively (Figure 6A).

First, we determined whether Dux mediates RA dependent *Zscan4* activation. Dux wild type (Dux^+/+^) and knock out (Dux^−/−^) ESCs (De Iaco et al., 2017) were cultured with or without VitA. In these cell lines, RA induction of *Zscan4* is completely impaired (Figure 6B) confirming, as expected, that Dux is necessary for RA-dependent ESCs to 2C-like transition. Second, to further evaluate our hypothesis, we generated knock-in recombinant ESCs in which Duxbl1-Flag could be overexpressed using by doxycycline-inducible (DOX) knock-in ESC line (Iacovino et al., 2014). The genetic construct and the strategy used for the knock-in cell line generation are reported in Figure 6C (top) and Figure S6A. The induction efficiency of Duxbl1 upon DOX treatment was assessed by western blot (Figure S6B). We evaluated the effects of *Duxbl1* overexpression on 2C state associated gene signature with or without retinoids. The analyses showed that the induction of *Duxbl1* without VitA leads to the reduction only of *Eif1a* and *Duxbl1;* however in the presence of VitA, *Duxbl1* ectopic expression downregulated *Zscan4* and, among others, MERV-L genes, thus reflecting a reduction of 2C-like state (Figure 6C, bottom). Considering *Zscan4* as ESCs to 2C-like transition bona fide marker, we checked whether *Duxbl1* inactivates *Zscan4* through direct binding to DBM by quantitative ChIP (qChIP) analysis (Figure S7A). Our data showed an enrichment of DBM fragments after anti-Flag immunoprecipitation in ESCs DOX^+^ cells (Figure 6D, top), compared to the IgG negative control and to a furthest region from DBM on *Zscan4* promoter (Figure S7B). Overall, we observed that Duxbl1 binding to the *Zscan4* promoter was associated to the reduced presence of H3 acetyl (Figure 6D, top). Consistently, RA led decreasing of Duxbl1 binding on *Zscan4* promoter and a resultant increasing of open chromatin conformation (Figure 6D, bottom).

## Discussion

In ESCs culturing systems, RA displays mainly a dual role. On one hand, it inhibits LIF signaling, which is required for stemness condition (Tighe and Gudas, 2004; Gudas and Wagner, 2011) and drives the up-regulation of genes involved in the differentiation process (Engberg et al., 2010). On the other hand, retinoids counteract the differentiation and maintain the pluripotency, mainly through the activation of WNT signaling (Wang et al., 2008), *Nanog* (Chen et al., 2007), and Phosphoinositide 3-kinase (PI3K) (Chen and Khillan, 2010).

Recent evidence reports that RA activates ZGA molecular signature (Tagliaferri et al., 2016) that is accompanied by the transition of ESCs to a high pluripotency state so-called 2C-like cells (Napolitano et al., 2019).

Here, we showed that without retinoids *Zscan4* spontaneous population is less than 1% of the whole ESCs population. Retinoids are able to activate the mitotic division of such population that promptly transit to 2C-like state activating MERV-L genes.

To elucidate the molecular network regulating ESCs to 2C-like transitions mediated by RA, we reconstructed Zscan4 global regulatory network by employing a reverse engineering approach on combined sequence data of known binding sites with ESCs expression datasets. Integrating these inferred findings with our experimental evidence, we found that RA promotes the Zscan4^+^ intermediates transition by co-regulating the expression of two members of Dux family: *Dux* and *Duxbl1*. Interestingly, our data showed that both *Dux* and *Duxbl1* are specifically expressed in Zscan4^+^ while, known 2C-like regulators, among others *Dppa2, Dppa4* although play a crucial role in the 2C state activation, do not significantly change between Zscan4^+^ and Zscan4^−^ state and may act in a broad range network (data not shown).

Based on the protein domain of Dux and Duxbl1, we propose that both molecules compete for Zscan4 proximal promoter binding via their N-terminal Hox portion, but only Dux can promote *Zscan4* transcription via its C-terminal transactivation region. Notably, we show that *Dux* is required for RA dependent transition of ESCs to 2C-like state, while *Duxbl1* counteracts such transition by direct downregulation of *Zscan4* expression. It would be interesting to evaluate whether Duxbl1 directly inactivate Dux targets, among others MERV-L, suggesting a potential role in balancing Zscan4 intermediates fluctuation. Remarkably, ectopic overexpression of *Duxbl1* strongly downregulates endogenous expression of *Duxbl1* but not *Dux*. This could be a negative feedback loops act to break the balance of 2C-like transition.

It is known that retinoids are crucial during preimplantation and embryonic development (Hofmann and Eichele, 1994; Huang et al., 2003), however, their effect on early cleavage has not been examined yet. Our data could be taken in consideration to study zygotic genome activation during both *in vivo* and *in vitro* development.

Another interesting consideration about our finding derives from the similar consensus binding motifs between Dux and its human ortholog DUX4 (Wu et al., 2010; Eidahl et al., 2016). Like Dux, DUX4 is involved in the positive regulation of ZSCAN4, PRAME, TRIM, MBD3L2, RFPL1, KHDC1, and FAM90 families (Geng et al., 2012). Aberrant DUX4 up-regulation is associated with Facio-Scapulo-Humeral Dystrophy (FSHD), and it alters the muscle transcriptome, splicing and differentiation (Mitsuhashi et al., 2018). It would be interesting to investigate whether the human ortholog of Duxbl1 could counteract DUX4 in FSHD and, thus, laying the basis for targeted therapy of such disease.

## Data Availability Statement

The raw data supporting the conclusions of this article will be made available by the authors, without undue reservation, to any qualified researcher.

## Author Contributions

GF designed the study and performed the data analysis and the interpretation with FV, IC, and AC. Bioinformatic analysis was performed by MC, LC, and TN. The experimental procedures were performed by DT, PM, MA, VR, SR, and TA. PM and GF wrote the manuscript with input from all authors and a critical reading from MD and LD. GF supervised all the experiments.

### Conflict of Interest

The authors LD and FV were employed by company CEINGE Biotecnologie Avanzate s.c.ar.l. The remaining authors declare that the research was conducted in the absence of any commercial or financial relationships that could be construed as a potential conflict of interest.
