# Supplementary material for: Retinoic Acid Induces Embryonic Stem Cells (ESCs) Transition to 2 Cell-Like State Through a Coordinated Expression of Dux and Duxbl1
Source: Front Cell Dev Biol. 2020 Jan 17;7:385. doi: 10.3389/fcell.2019.00385 (PMC6979039; doi:10.3389/fcell.2019.00385)
Supplement: Supplementary file 1 [file Data_Sheet_1.pdf]

Supplementary Material

Figure S1. RA induces Zscan4 protein expression in ESCs.

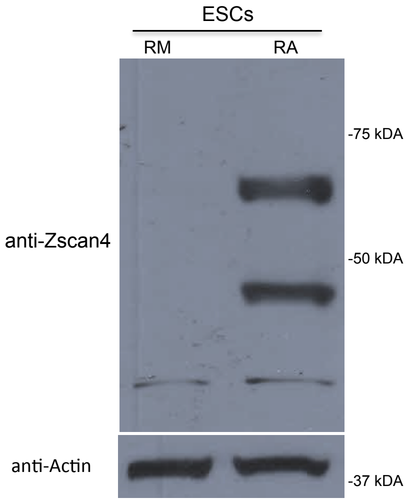

ESCs were cultured with 1.5 µM RA for 72h. Following the treatment Zscan4 protein expression was assessed by Western blot analysis.

Figure S2. RA signalling is required for Zscan4 induction.

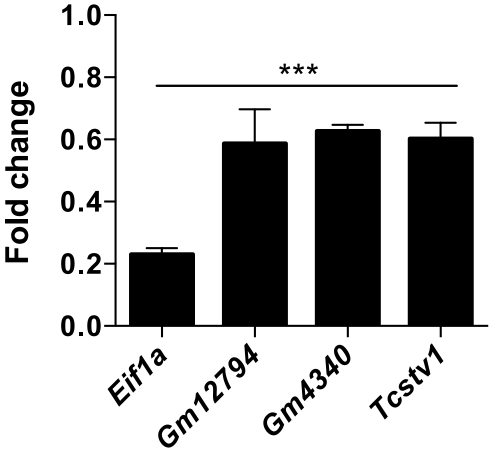

ESCs cells were cultured in VitA^{minus} condition. Following treatment, *Eif1a*, *Gm12794*, *Gm4340* and *Tcstv1* expression levels were analysed by qPCR. The average and SEM of all the experiments were performed on the duplicate samples from three independent biological experiments and is shown: ***, p < .001, in a Student's *t* test.

Figure S3 Validation of Zscan4_LNGFR ESCs line

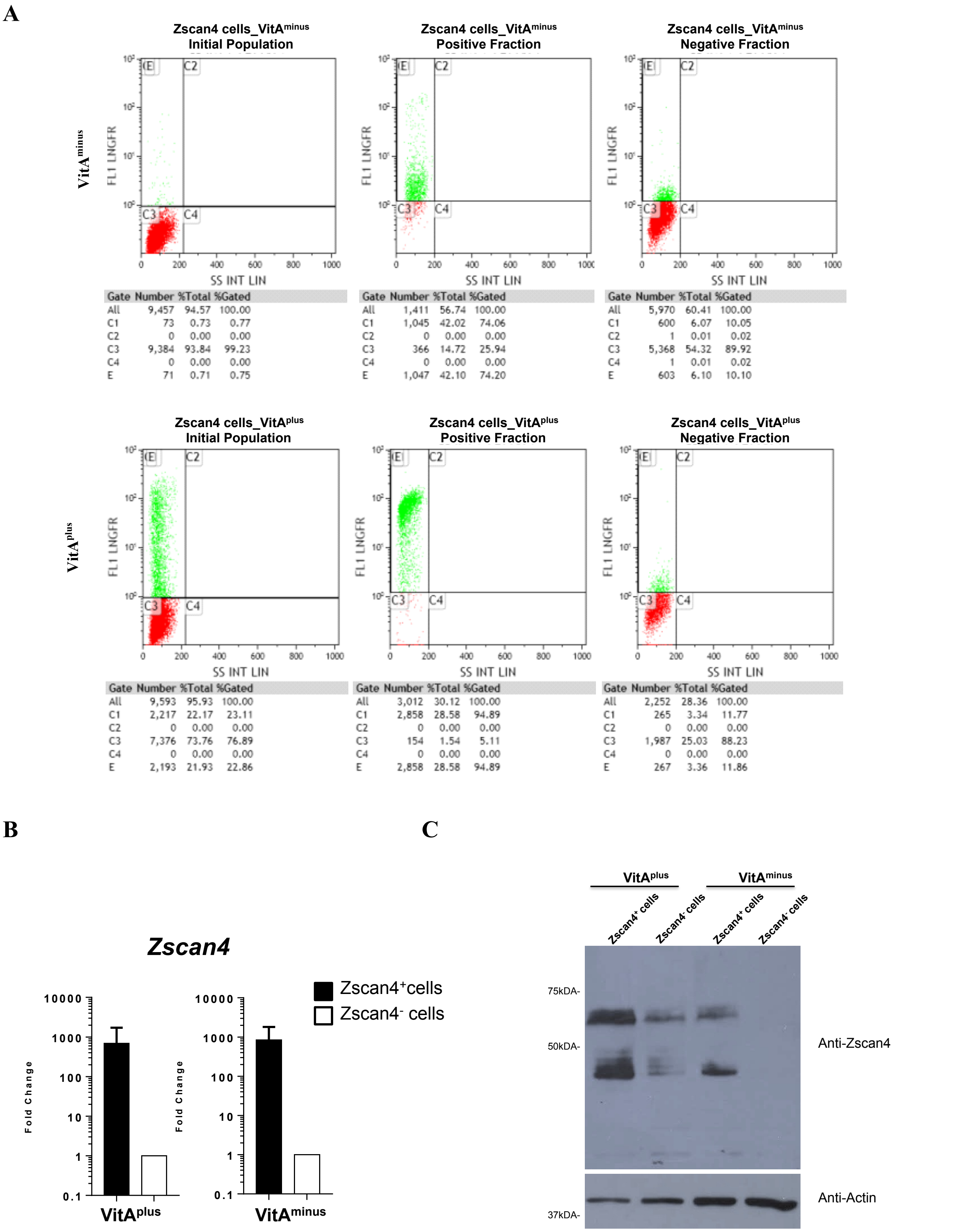

ESCs were cultured in VitA^{plus} medium for 72h. After the treatment the cells were immunomagnetically separated through AutoMACs proseparator (Miltenyi Biotec) and analyzed by cytofluorimetry to validate the efficiency of separation (A). Furthermore, qPCR (B) and western blot analyses (C) were performed to confirm the Zscan4 enrichment in LNGFR positive cell fraction.

Supplementary video

The video describes a time lapse of ESCs expressing GFP under the control of Zscan4 promoter. Upon RA treatment, Zscan4 induction leads to an increase of fluorescent cells.

Figure S4. Global regulatory network from a collection of ESCs gene expression profiles

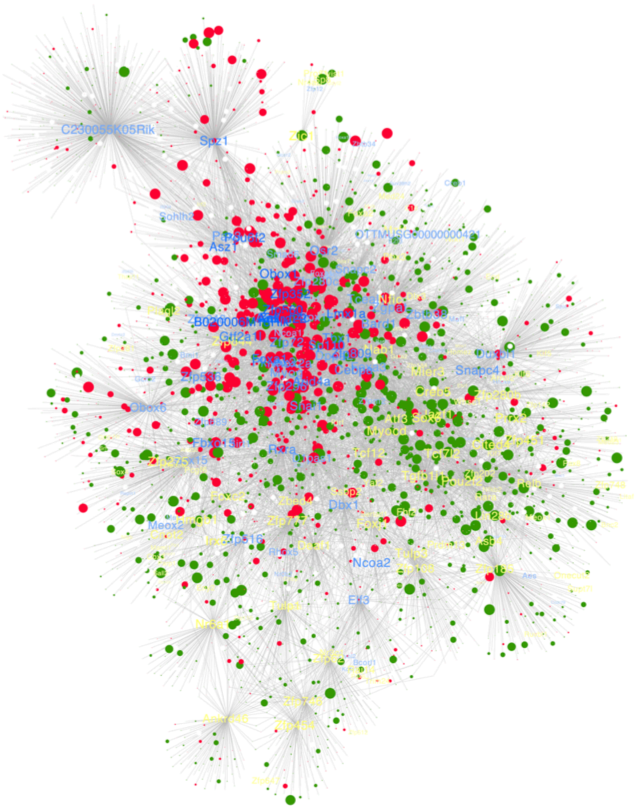

Global gene network reconstructed with an information theoretical inference approach from a collection of 754 mouse ESCs specific gene expression profiles. An over-expression in RA-Zscan4^{+} is showed in red compared to RA-Zscan4^{-} which is showed in green.

Figure S5. Duxbl1 binds Zscan4 promoter.

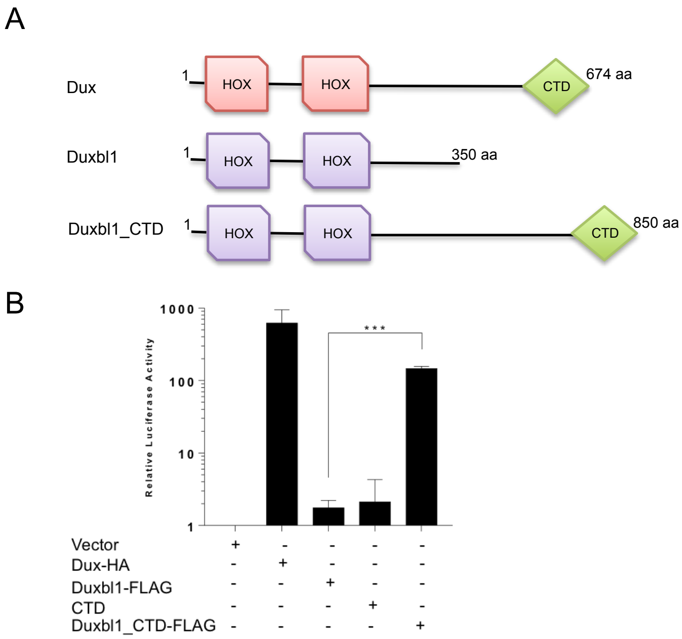

A. The transactivation domain, CTD, placed at C-term of Dux, was added to Duxbl1 protein. **B.**HEK293T cells with Luciferase-reporter gene under Zscan4 promoter regulation were transiently transfected with: control Vector, or Dux-HA, Duxbl1-Flag or with Duxbl1_CTD-Flag. Data shown represent relative luciferase activity normalized against β-galactosidase activity. The average and SEM of all the experiments were performed on three independent biological experiments and are shown: **, $p < .01$, ***, $p < .001$, in a Student's *t* test.

Figure S6. ESC$^{Duxbl1\text{-}Flag}$ transgenic cell lines.

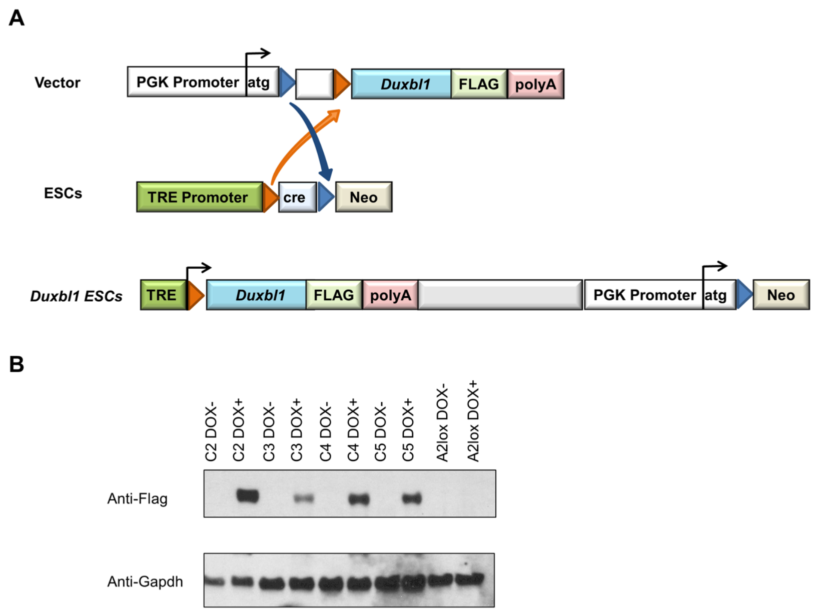

(A) Schematic representation of construct Duxbl1-Flag, DOX-inducible, used for ESCs transgenic lines generation. (B) Western blot assay of ESC$^{Duxbl1\text{-}Flag}$ cells DOX-inducible for system validation, using anti-Flag and anti-Gapdh antibodies.

Figure S7. *Duxbl1* ectopic characterization.

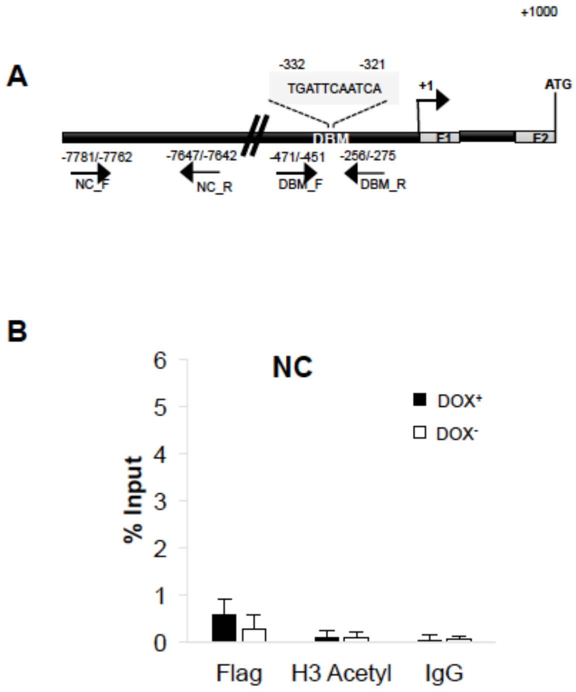

(**A**) Schematic Zscan4 sequence of the putative DBM (within the previously identified RA responsive region) and primers position used for ChIP assay are indicated. (**B**) Duxbl1 not binds Zscan4 promoter away from DBM. ES$^{Duxbl1\text{-}Flag}$ cells were cultured with or without DOX for 72 hours and analysed by ChIP assay as Negative control of ChIP experiment indicated in Figure 6D. The average and SEM of all the experiments were performed on three independent biological experiments and are shown: **, p < .01***, p < .001 in a Student's *t* test.

Table S1. Analysis of Canonical RARE motifs

DR-type	sequence name	start	end	strand	p-value	q-value	matched sequence
DR-5	Gm13119	1716	1732	+	9,13E-07	0,099	AGTTCAAATTTAGGTCA
DR-2	Dnah9	1130	1143	-	8,92E-07	0,0716	AGTTCATGAGTTCA
DR-5	BC080695	1725	1741	+	8,82E-07	0,099	AGTTCAAACTTAGGTCA
DR-1	Crabp2	618	630	-	8,16E-06	0,078	AGGGCAGAGGTCA
DR-5	Cyp26a1	15	31	-	7,77E-07	0,004	AGTTCACCCAAAGTTCA
DR-2	Got2	1349	1362	+	7,68E-07	0,0986	AGGTCATGAGTTCA
DR-2	Mest	2270	2283	-	7,68E-07	0,0716	AGTTCAGAGGTTCA
DR-1	Tmem189	2247	2259	-	6,84E-07	0,0731	AGGTCAAAGGTCA
DR-1	Prex2	1734	1746	-	6,84E-07	0,0731	AGGTCATAGGTCA
DR-1	Ino80c	62	74	+	6,84E-07	0,0731	AGGTCAAGGTTCA
DR-2	Crabp2	1127	1140	+	5,14E-07	0,005	AGTTCACCAGGTCA
DR-2	B020004J07Rik	2386	2399	+	5,14E-07	0,0825	GGTTCAGAGGTTCA
DR-2	AA986860	2382	2395	+	5,14E-07	0,0825	GGTTCAGAGGTTCA
DR-2	Tdpoz5	445	458	-	5,14E-07	0,0825	AGGTCAGGAGTTCA
DR-2	Il17rd	476	489	-	5,14E-07	0,0716	AGGTCAGGAGTTCA
DR-5	Rarb	15	56	+	5,00 e-07	0,003	GGTTCACCGAAAGTTCA
DR-5	Csn3	405	421	-	4,62E-08	0,011	GGGTCACCTGCAGGTCA
DR-5	Mest	2353	2369	-	3,49E-07	0,0414	GGGTCAGAGGAAGTTCA
DR-3	Drd2	314	328	-	2,95E-05	0,113	GGGTCACCCTGGCCA
DR-2	Mreg	1270	1283	+	2,36E-07	0,0825	GGTTCAGAGGGTCA
DR-2	Hoxb1	1629	1642	-	1,65E-05	0,081	AGGGCAAGAGTTCA
DR-2	Stra8	300	287	+	1,65E-05	0,019	GGGTGAAAAGGTCA
DR-5	Prkch	598	614	-	1,53E-07	0,05	GGGTCATCCCAGGTTCA
DR-3	Csn3	416	430	-	1,41E-07	0,017	AGGTCACCAGGGTCA
DR-3	Ackr3	1645	1659	+	1,41E-07	0,017	AGGTCAGGCAGGTCA
DR-1	Apol7b	1183	1195	-	1,21E-07	0,0259	GGGTCAAGGGTCA
DR-1	1700109H08Rik	981	993	+	1,21E-07	0,0259	GGGTCACAGGTCA
DR-1	4930488B22Rik	52	64	-	1,21E-07	0,0259	GGGTCAAGGGTCA
DR-1	Pla2g2a	446	458	+	1,21E-07	0,0293	AGGTCAGGGGTCA

Table S2. List of Master Regulators

Master Regulator	***p.value***	***FDR***	***ES***	***NES***	***size***
Zfp560	0.000183385	0.0041021	0.86564392	2.85603522	74
Ankrd22	0.000179986	0.0041021	0.85853053	2.88472886	81
Zfp352	0.000167504	0.0041021	0.85120576	3.15650269	158
B020006M18Rik	0.000186359	0.0041021	0.8442614	2.71178247	62
Obox1	0.000164447	0.0041021	0.81211733	3.04726253	173
Zscan4c	0.000149388	0.0041021	0.7921011	3.24156322	366
Lmx1a	0.000161891	0.0041021	0.77326238	2.95387844	200
Pou6f2	0.000173581	0.0041021	0.77167242	2.72727753	112
Asz1	0.000169062	0.0041021	0.76379968	2.79472032	144
Nelfa	0.000157803	0.0041021	0.75377359	2.95227565	244
Csrnp2	0.00015873	0.0041021	0.71367173	2.77490062	228
Foxa1	0.000169062	0.0041021	0.7123334	2.60640675	144
Sp110	0.000150353	0.0041021	0.69376765	2.81941616	344
Gtf2a1l	0.000161316	0.0041021	0.68360789	2.61853141	204
Zfp809	0.000163613	0.0041021	0.65783622	2.4885116	186
Spz1	0.00014941	0.0041021	0.65055644	2.66626808	372
Arid4a	0.000168776	0.0041021	0.64776656	2.37596137	147
Tbx1	0.000165481	0.0041021	0.6385288	2.39049067	171
Fbxo15	0.000159413	0.0041021	0.60573206	2.34786192	223
Pknox2	0.000146284	0.0041021	0.5958041	2.48806773	457
Arnt	0.000147645	0.0041021	0.59138646	2.45119244	423
Zfp296	0.00015674	0.0041021	0.58773012	2.31359437	257
Cebpa	0.000156104	0.0041021	0.58711144	2.31775664	263
Ncoa2	0.000173641	0.0041021	0.57593693	2.03057978	110
Osr2	0.000143761	0.0041021	0.56526973	2.38700017	522
Zfp72	0.000158053	0.0041021	0.56488081	2.20516775	236
Rhox2a	0.000166306	0.0041021	0.56154884	2.0985916	169
Snai1	0.000159949	0.0041021	0.56032203	2.16747494	221
Rxra	0.00015425	0.0041021	0.5598233	2.2357033	294
Snapc4	0.000355872	0.00417009	0.55688842	1.90430096	90
Zfp536	0.000161812	0.0041021	0.55040094	2.10042728	196
Bard1	0.000160282	0.0041021	0.54187492	2.08437045	209
Dbx1	0.000168407	0.0041021	0.53306351	1.97024959	156
Myog	0.000166667	0.0041021	0.5129365	1.91451141	167
Meox2	0.000501672	0.00527044	0.49708232	1.84982991	163
Dppa2	0.000152253	0.0041021	0.495051	1.99715803	323
Zfp516	0.001028454	0.00800898	0.48724074	1.7645557	135
Tceal1	0.000667111	0.00622184	0.48700516	1.81642273	166
Tfap2a	0.000152858	0.0041021	0.485972	1.95555289	315
Zbtb38	0.000162101	0.0041021	0.48590201	1.8569527	201
Zgpat	0.000155521	0.0041021	0.48362188	1.91666275	272
C230055K05Rik	0.000137722	0.0041021	0.48047309	2.07226326	678
Foxa3	0.000156814	0.0041021	0.47465759	1.8666114	251
Sox15	0.000154536	0.0041021	0.47016241	1.8778858	296

Obox6	0.000159923	0.0041021	0.46963045	1.8174395	222
Spib	0.000490838	0.00522575	0.46695637	1.76643713	186
Zfp280c	0.000149321	0.0041021	0.46458689	1.89746009	359
Sox17	0.000318573	0.0041021	0.46410743	1.79434199	218
Thrb	0.000324992	0.0041021	0.45433653	1.73294979	197
D1pas1	0.000153681	0.0041021	0.45206913	1.80953137	300
Duxbl1	0.000156104	0.0041021	0.44460685	1.855187	263
Sohlh2	0.000151722	0.0041021	0.43651036	1.76459462	328
Elf3	0.000476493	0.00515976	0.43228473	1.835257	234
Gtf2e1	0.000784683	0.00699708	0.42717921	1.67875976	250
Aire	0.000140706	0.0041021	0.42603938	1.81671308	584
Trerf1	0.001107069	0.00845357	0.42178257	1.65063516	243
Mtf2	0.000462677	0.00515976	0.4119236	1.64164384	286
Rhox12	0.001237241	0.00886932	0.40473324	1.60974161	283
Rhox5	0.00013113	0.0041021	0.40351181	1.77506801	906
Fgfbp1	0.000919118	0.00746096	0.39634356	1.58766996	301
Zfp689	0.000448029	0.00511873	0.39092599	1.59558682	356
Zfp790	0.000915751	0.00746096	0.38844442	1.56361027	317
Tbx3	0.000133032	0.0041021	0.38828628	1.69953094	828
Vav1	0.00089579	0.0074432	0.38440527	1.56728572	353
Zfpm2	0.00043573	0.00505134	0.36732206	1.54136873	486
Egr2	0.000133583	0.0041021	0.36366942	1.58927479	803
Carf	0.000117385	0.0041021	0.30040783	1.37134759	1837
Npm1	0.001311762	0.0093668	-0.2653889	-1.2797224	963
Rcor1	0.000825764	0.00710457	-0.2739702	-1.3140841	873
Tcf15	0.000439367	0.00505134	-0.2746396	-1.3264977	981
Wwtr1	0.001203369	0.00870762	-0.2795066	-1.3364423	822
Cnbp	0.001342282	0.00943981	-0.2827527	-1.4384202	3477
Hdac11	0.00034638	0.0041021	-0.3147684	-1.4667773	589
Hey1	0.000944287	0.00757086	-0.3150081	-1.4401608	456
Med27	0.000926498	0.00746096	-0.3165394	-1.4353694	413
Aatf	0.000343761	0.0041021	-0.3167918	-1.4711386	567
Smad5	0.000926212	0.00746096	-0.3176136	-1.4397752	411
Zmynd8	0.000331895	0.0041021	-0.3177115	-1.4691002	527
Zfp322a	0.000638978	0.00599001	-0.317837	-1.4596309	480
Pou3f1	0.001209921	0.00870762	-0.3180454	-1.4270876	375
Pou3f3	0.000324465	0.0041021	-0.3202207	-1.4744853	497
Cebpb	0.000926212	0.00746096	-0.3204151	-1.4524746	411
Polr2i	0.000625195	0.00589102	-0.3266667	-1.486489	434
Ankrd52	0.000919963	0.00746096	-0.3275477	-1.4845961	409
Zkscan8	0.000324465	0.0041021	-0.3285312	-1.5124593	496
Zbtb4	0.000347826	0.0041021	-0.3294438	-1.5395974	609
Hmga2	0.00062054	0.00587745	-0.3318477	-1.5073136	421
Sall3	0.001207365	0.00870762	-0.3357698	-1.500549	360
Usf1	0.000346861	0.0041021	-0.3400885	-1.5843645	588
Ahrr	0.00120012	0.00870762	-0.3403981	-1.5146452	346

Nkrf	0.000608088	0.0057895	-0.3407837	-1.5286167	374
Zfp770	0.000907716	0.00746096	-0.3423367	-1.5269018	354
Pitx2	0.000334001	0.0041021	-0.3426162	-1.584426	531
Ruvbl1	0.000345781	0.0041021	-0.3441332	-1.6017514	583
Gli3	0.000309693	0.0041021	-0.3445926	-1.5657926	423
Gcfc2	0.000314663	0.0041021	-0.345368	-1.5725102	438
Zfp606	0.001139277	0.0085004	-0.3454963	-1.5146305	298
Tead3	0.000327654	0.0041021	-0.3461714	-1.5976935	514
Lbh	0.001444669	0.00996549	-0.3465619	-1.5267036	313
Sox10	0.000303582	0.0041021	-0.3477813	-1.5654283	385
Cbfa2t2	0.00113701	0.0085004	-0.3478021	-1.5199407	287
Hmg20a	0.000300933	0.0041021	-0.3494509	-1.5625211	361
Dlx4	0.001132824	0.0085004	-0.3501141	-1.5351744	296
Phtf1	0.000336247	0.0041021	-0.3502734	-1.6211074	537
Atf7	0.000591541	0.00566145	-0.3508995	-1.5591926	339
Smad3	0.000302663	0.0041021	-0.3511533	-1.5653923	353
Phox2a	0.001451379	0.00997414	-0.3512125	-1.5433043	306
Cdyl	0.001398601	0.00975818	-0.3550445	-1.5388442	267
Zfp444	0.000300481	0.0041021	-0.3553111	-1.5832919	351
Ikzf2	0.001136041	0.0085004	-0.3554481	-1.5581758	297
Zfp281	0.001109878	0.00845357	-0.3562668	-1.5398311	260
Ankrd42	0.001156738	0.0085608	-0.3567961	-1.5779729	321
Hmga1	0.000344709	0.0041021	-0.3584988	-1.6678077	578
Dppa3	0.001342642	0.00943981	-0.3598342	-1.5260866	218
Vax1	0.001105278	0.00845357	-0.3599861	-1.5561679	259
Ckap5	0.000866802	0.00723522	-0.3600736	-1.5899861	318
Gabpb1	0.000326797	0.0041021	-0.3609975	-1.6657607	511
Zfp367	0.00107904	0.00832272	-0.3618728	-1.5433496	229
Wiz	0.001333333	0.00943981	-0.3621534	-1.5381396	221
Glis1	0.000321027	0.0041021	-0.362856	-1.6641148	474
Asb6	0.000302755	0.0041021	-0.3633703	-1.6357857	384
Msrb2	0.001360544	0.00952902	-0.3635246	-1.555034	236
Rora	0.000809935	0.00710457	-0.3638467	-1.5527703	230
Lmo1	0.001119194	0.00848916	-0.3650424	-1.5818739	266
Whsc1	0.000831716	0.00710457	-0.3653321	-1.5753449	254
Irf1	0.000580552	0.00558552	-0.3658325	-1.6075479	306
Pawr	0.000810373	0.00710457	-0.3664195	-1.56116	228
Crxos	0.000319489	0.0041021	-0.3665357	-1.683274	480
Gbx1	0.00057971	0.00558552	-0.3679344	-1.623892	317
Atf1	0.001045205	0.00809591	-0.3683806	-1.5520542	202
Nfxl1	0.000322581	0.0041021	-0.3689366	-1.6981778	494
Zc3h8	0.001324153	0.00941849	-0.3695989	-1.5602055	207
Psmc3	0.000827586	0.00710457	-0.3701048	-1.5936262	251
Sox13	0.000857633	0.00719153	-0.3709152	-1.6221687	292
Smarcb1	0.000856653	0.00719153	-0.371071	-1.6225866	290
Eny2	0.00029753	0.0041021	-0.3711622	-1.6500817	343

Batf3	0.001029601	0.00800898	-0.3712534	-1.5552624	190
Btaf1	0.000857633	0.00719153	-0.3715485	-1.6249384	292
Msx3	0.000852031	0.00719153	-0.372477	-1.6328252	297
Sp8	0.000826674	0.00710457	-0.3737249	-1.6066309	247
Arid3a	0.000303582	0.0041021	-0.3738895	-1.6829464	385
Sap30bp	0.000544514	0.00549929	-0.3739096	-1.6007113	237
Supt16	0.000535045	0.00546404	-0.3747789	-1.5949016	225
Klf6	0.00030175	0.0041021	-0.3753557	-1.6700112	347
Kdm5b	0.000826674	0.00710457	-0.376026	-1.6173042	248
Tbl1x	0.000302206	0.0041021	-0.3773066	-1.6849108	358
Klf10	0.000779626	0.00699708	-0.3784438	-1.5905497	197
Pax8	0.000826674	0.00710457	-0.3792984	-1.6360942	253
Bdp1	0.001028278	0.00800898	-0.3794764	-1.5828248	186
Eid1	0.000783699	0.00699708	-0.3796166	-1.5910883	194
Zfp637	0.000525901	0.00546149	-0.379721	-1.6011232	204
Zfp354c	0.000288934	0.0041021	-0.3812487	-1.6795088	313
Zfp763	0.000342583	0.0041021	-0.3815229	-1.768235	551
Msl3	0.000828043	0.00710457	-0.3821614	-1.6508772	256
Zkscan14	0.000542446	0.00549929	-0.3825204	-1.6381184	238
Gatad1	0.001024066	0.00800898	-0.3827828	-1.5959492	185
Tcf7	0.000783699	0.00699708	-0.3835216	-1.6074553	194
Tcfl5	0.00056101	0.00552309	-0.3851442	-1.6703418	269
Zfp30	0.000288934	0.0041021	-0.3853158	-1.6920418	304
Meis3	0.000564653	0.00552309	-0.3857185	-1.678246	277
Fhl2	0.0003427	0.0041021	-0.3861249	-1.7927147	562
Skp1a	0.000768246	0.00698683	-0.3871843	-1.6055396	178
Dlx3	0.000528821	0.00546149	-0.3875968	-1.6347724	205
Satb1	0.000294291	0.0041021	-0.3892142	-1.7293991	336
Zfp764	0.00056802	0.00552309	-0.3905858	-1.7073871	288
Zfp53	0.000532907	0.00546404	-0.3910546	-1.6544649	213
Clock	0.000565931	0.00552309	-0.3912352	-1.7042857	280
Wtip	0.001020408	0.00800898	-0.3915727	-1.6278194	181
Yaf2	0.000317259	0.0041021	-0.3923946	-1.7938069	460
Asb8	0.00056802	0.00552309	-0.3925447	-1.7159503	288
Etv4	0.000562114	0.00552309	-0.3926924	-1.7037314	270
Zfp202	0.000565611	0.00552309	-0.3928921	-1.714969	283
Thrap3	0.000259875	0.0041021	-0.3941092	-1.6563897	197
Zscan21	0.000510595	0.00533353	-0.3942926	-1.630262	175
Ikzf4	0.0002574	0.0041021	-0.3954395	-1.6565831	190
Per2	0.00027027	0.0041021	-0.3959626	-1.70037	240
Zfp236	0.000270124	0.0041021	-0.3986381	-1.6984302	228
Pbx3	0.000310078	0.0041021	-0.398711	-1.8076258	415
Meis2	0.000259202	0.0041021	-0.3998661	-1.6824379	198
Zeb2	0.000992063	0.00788475	-0.4005172	-1.6409216	162
Cers2	0.000289519	0.0041021	-0.4008288	-1.7604006	305
Bach2	0.000280112	0.0041021	-0.400853	-1.7433893	275

Dip2a	0.000255363	0.0041021	-0.4016206	-1.6719963	183
Mef2a	0.000286123	0.0041021	-0.4016815	-1.7629448	300
Hsf5	0.000257003	0.0041021	-0.4034952	-1.6848737	187
Btg2	0.000321543	0.0041021	-0.403505	-1.8480446	470
Med20	0.000265534	0.0041021	-0.4038901	-1.7048881	208
Zfp341	0.000275482	0.0041021	-0.4044651	-1.7411427	250
Tada2a	0.000304878	0.0041021	-0.4047344	-1.8297567	398
Taf9	0.000275482	0.0041021	-0.4061764	-1.7485094	250
Nkx6-2	0.00049456	0.00522575	-0.4097693	-1.6733895	159
Etv2	0.000261643	0.0041021	-0.4105843	-1.7234355	196
Mycbp	0.000259067	0.0041021	-0.4113011	-1.7238701	192
Bhlhe40	0.000300481	0.0041021	-0.4117436	-1.8411895	362
Nr3c1	0.001201057	0.00870762	-0.4143205	-1.6485029	133
Tbpl1	0.000245038	0.0041021	-0.4160157	-1.6955831	155
Cited2	0.000493097	0.00522575	-0.4184706	-1.7008574	153
Tsc22d4	0.00026441	0.0041021	-0.4194408	-1.7690815	205
Hivep1	0.000728863	0.00669434	-0.4219729	-1.6905546	140
Mzf1	0.001412429	0.00979615	-0.4237466	-1.6451664	115
Pou5f1	0.001414761	0.00979615	-0.4245918	-1.6396193	112
Kcnip3	0.000279486	0.0041021	-0.4245941	-1.8456456	273
Pias2	0.000957854	0.0076461	-0.4247238	-1.6866251	132
Prdm8	0.000270416	0.0041021	-0.4248751	-1.8152042	233
Hmgb2	0.000261506	0.0041021	-0.4268574	-1.7993384	203
Rai14	0.000248016	0.0041021	-0.4278188	-1.7527764	162
Fos	0.000247097	0.0041021	-0.4282642	-1.7466368	157
Zfp707	0.000732422	0.00669434	-0.42922	-1.7223567	142
Six1	0.000304044	0.0041021	-0.4298751	-1.9282441	374
Rad54l2	0.000717875	0.0066613	-0.430156	-1.7031085	129
Zfp646	0.001175641	0.00866561	-0.4303005	-1.6595653	111
Yeats2	0.000290192	0.0041021	-0.430675	-1.9045433	322
Tanc2	0.000722717	0.00667236	-0.4331736	-1.7257675	134
Ascc1	0.001183712	0.00869006	-0.4334561	-1.696574	121
Preb	0.000252589	0.0041021	-0.4336451	-1.7876518	171
Cnot2	0.000479846	0.00515976	-0.433742	-1.7328761	135
Ilf2	0.00047081	0.00515976	-0.4354731	-1.6906941	115
Relb	0.000255297	0.0041021	-0.4356855	-1.801407	175
Zfp182	0.000272851	0.0041021	-0.4357678	-1.8729535	244
Hdac7	0.000282805	0.0041021	-0.4369744	-1.9073878	283
Ankrd46	0.000259875	0.0041021	-0.4373562	-1.8381512	197
Plagl2	0.000255037	0.0041021	-0.4380566	-1.8087628	173
Rest	0.000272554	0.0041021	-0.4383517	-1.8852166	245
Stat1	0.001152074	0.0085608	-0.4388701	-1.6573139	97
Meis1	0.000479846	0.00515976	-0.4389384	-1.7328981	127
Ccnt2	0.000261233	0.0041021	-0.4391386	-1.8405632	194
Zfp593	0.000247525	0.0041021	-0.4396989	-1.7985044	160
Zfp618	0.00026976	0.0041021	-0.4446111	-1.8962198	229

Asb4	0.000479386	0.00515976	-0.4458411	-1.7666752	130
Nab1	0.000246245	0.0041021	-0.4477259	-1.8160878	151
Lin28b	0.00047081	0.00515976	-0.44797	-1.7219438	109
Fem1c	0.000461681	0.00515976	-0.4483994	-1.7076465	102
Zfp422	0.000238095	0.0041021	-0.4491685	-1.761334	123
Jun	0.000261506	0.0041021	-0.4515832	-1.9035655	203
Deaf1	0.000239406	0.0041021	-0.4519154	-1.7765135	125
Pura	0.000255363	0.0041021	-0.4524316	-1.8835287	183
Atf3	0.000302572	0.0041021	-0.4526012	-2.018707	354
Tulp3	0.000471587	0.00515976	-0.4544326	-1.7548539	112
Tulp1	0.000241721	0.0041021	-0.4562659	-1.8266665	138
Sowahc	0.000248016	0.0041021	-0.4565787	-1.8618031	158
Zfp62	0.00023084	0.0041021	-0.4583759	-1.7456404	102
Kat5	0.000233372	0.0041021	-0.4590042	-1.7597549	107
Hmgb1	0.00023084	0.0041021	-0.4634861	-1.7530237	98
Zfp185	0.000266738	0.0041021	-0.4638876	-1.9660672	215
Zfp746	0.000233372	0.0041021	-0.467024	-1.7905014	107
Foxj1	0.000238095	0.0041021	-0.4692155	-1.8399448	123
Tcf12	0.000267094	0.0041021	-0.4792172	-2.0341031	219
Dmrtb1	0.000235405	0.0041021	-0.4804951	-1.8469667	109
Zfp280b	0.000234962	0.0041021	-0.481801	-1.8834243	119
Rbfox2	0.000234028	0.0041021	-0.4834656	-1.8570864	108
Tcf7l2	0.000439271	0.00505134	-0.4841321	-1.7461745	73
Tgfb1i1	0.000301841	0.0041021	-0.485418	-2.1693245	360
Zfp454	0.000452694	0.0051399	-0.4862062	-1.783513	82
Mier3	0.00024728	0.0041021	-0.4875338	-1.9909591	159
Zfp777	0.000266738	0.0041021	-0.4950593	-2.1049339	222
Nfic	0.000240906	0.0041021	-0.4963326	-1.9860504	137
Zfp644	0.000241721	0.0041021	-0.4982898	-1.9949097	138
Pou2f2	0.000247341	0.0041021	-0.5065967	-2.0741214	161
Jazf1	0.000235128	0.0041021	-0.5084544	-1.9609859	111
Prrx2	0.000240906	0.0041021	-0.5119004	-2.048344	137
Nr6a1	0.000239406	0.0041021	-0.5166833	-2.0311208	125
Myocd	0.000239464	0.0041021	-0.5265972	-2.0911756	132
Cited4	0.000218531	0.0041021	-0.5330536	-1.9187099	72
Sox6	0.000259808	0.0041021	-0.5409714	-2.2770629	199
Zfp451	0.000224921	0.0041021	-0.5539263	-2.0308343	81
Creb5	0.000248633	0.0041021	-0.5541833	-2.2709822	163

Table S3. Zscan4c Promoter analysis

pattern name	*start*	*end*	*normalized binding score*	*p.value*	*matched sequence*
Bach2	-1424	-1417	0.78658	0.002	GTCATGTG
Duxbl1	-332	-322	0.70539	3.00E-05	TGATTCAATCA
Elf3	-553	-541	0.79535	2.00E-05	AACCAGGAAATTT
Fos	638	644	0.81653	0.00012	TGACACA
Fos	-1773	-1767	0.75251	0.00031	TGCCTCA
Fos	601	607	0.71219	0.00037	TGAAACA
Fos	-1485	-1479	0.70509	0.00043	TGAGTCA
Fos	525	531	0.70509	0.00043	TGAGTCA
Foxa3	-878	-866	0.91544	0	AAAAAACAAACAA
Hmga1	-1291	-1285	0.92027	0.00012	AAAATAA
Hmga1	-2054	-2048	0.90217	0.00024	AAAATAG
Hmga1	-1633	-1627	0.90217	0.00024	AAAATAG
Hmga1	-1368	-1362	0.86281	0.00037	AAAATTT
Hmga1	-1343	-1337	0.86281	0.00037	AAAATTT
Hmga1	405	411	0.86281	0.00037	AAAATTT
Hmga1	-1062	-1056	0.80959	0.00061	AAAATTG
Hmga1	341	347	0.80819	0.00067	TAAATAT
Hmga1	-2059	-2053	0.77308	0.00079	TAAATAA
Hmga1	-2328	-2322	0.76587	0.00104	GAAATAC
Hmga1	-2157	-2151	0.76023	0.0011	TAAATTC
Hmga1	703	709	0.76023	0.0011	TAAATTC
Hmga1	-2163	-2157	0.72125	0.0014	GAAATAT
Hmga1	-1594	-1588	0.70664	0.00165	AAAAAAC
Hmga1	-1513	-1507	0.70664	0.00165	AAAAAAC
Hmga1	-878	-872	0.70664	0.00165	AAAAAAC
Hmga1	-862	-856	0.70664	0.00165	AAAAAAC
Hmga2	-1135	-1126	0.92373	0.00012	CTGGAATTAA
Hmga2	-320	-311	0.9079	0.00021	CAGGAAAAAA
Hmga2	-1521	-1512	0.89214	0.00034	TAGCAAAAAA
Hmga2	-1986	-1977	0.88168	0.00045	AACCAATAAA
Hmga2	170	179	0.87377	0.00057	TCACAATTAT
Hmga2	420	429	0.84749	0.00108	GCAGAATAAC
Hmga2	-1069	-1060	0.84744	0.00109	AGGGAAAAAA
Hmga2	-1556	-1547	0.84479	0.00117	TTGCATTAAT
Hmga2	-1349	-1340	0.83955	0.0013	ATGTAAAAAA
Hmga2	-883	-874	0.8343	0.00145	TTGTAAAAAA
Hmga2	677	686	0.82907	0.00159	CATTAATTAT
Hmga2	-866	-857	0.82903	0.00162	AAACAAAAAA
Hmga2	-1598	-1589	0.8264	0.0017	CATGAAAAAA
Hmga2	-550	-541	0.82632	0.00174	CAGGAAATTT
Hmga2	-1712	-1703	0.82113	0.00188	AGGGATTAAA
Hmga2	117	126	0.81846	0.002	GTGTATTTAT
Hmga2	195	204	0.81591	0.00204	GTTCAATATT

Hmga2	-2062	-2053	0.81586	0.00208	TTGTAAATAA
Hmga2	-1068	-1059	0.80278	0.00255	GGGAAAAAAA
Hmga2	-2166	-2157	0.80271	0.00261	GATGAAATAT
Hmga2	-777	-768	0.80003	0.00276	CCACAAATTT
Hmga2	-1545	-1536	0.79488	0.00292	CATTAATATA
Hmga2	674	683	0.79219	0.00309	TAACATTAAT
Hmga2	-1519	-1510	0.78962	0.00317	GCAAAAAAAA
Hmga2	-1295	-1286	0.78955	0.00322	AGGGAAAATA
Hmga2	-156	-147	0.78686	0.00337	ATGGATAAAA
Hmga2	-1294	-1285	0.78435	0.00343	GGGAAAATAA
Hmga2	-2165	-2156	0.78177	0.00356	ATGAAATATA
Hmga2	-2331	-2322	0.78167	0.00362	CCCGAAATAC
Hmga2	-1637	-1628	0.78165	0.00364	GAAGAAAATA
Hmga2	621	630	0.77897	0.00379	ACCCATAAAA
Hmga2	-99	-90	0.7738	0.00403	GTGAATTTAA
Hmga2	312	321	0.7738	0.00403	GTGAATTTAA
Hmga2	-1713	-1704	0.77361	0.0041	CAGGGATTAA
Hmga2	-2317	-2308	0.7712	0.00414	GGTTAAAAAA
Hmga2	-319	-310	0.7686	0.00425	AGGAAAAAAT
Hmga2	-1027	-1018	0.76855	0.00429	ACAGAAAAAC
Hmga2	-1548	-1539	0.76854	0.0043	ATTCATTAAT
Hmga2	-1518	-1509	0.76596	0.00443	CAAAAAAAAA
Hmga2	358	367	0.76585	0.00452	AAGTATTATT
Hmga2	-2343	-2334	0.76333	0.00459	TTTGAATATT
Hmga2	-1241	-1232	0.76319	0.00468	GAAGATAAAA
Hmga2	-2110	-2101	0.76088	0.0047	GGGGAATAAG
Hmga2	326	335	0.76059	0.00482	CCTGATTATT
Hmga2	-490	-481	0.75793	0.00502	GAAGATAAAT
Hmga2	527	536	0.7528	0.00523	AGTCAATTTT
Hmga2	-1978	-1969	0.75273	0.00528	AATGATTTAA
Hmga2	-1418	-1409	0.7527	0.00532	TGGCATTTTT
Hmga2	316	325	0.74493	0.00573	ATTTAATATT
Hmga2	-549	-540	0.74231	0.0059	AGGAAATTTT
Hmga2	-2061	-2052	0.73713	0.00624	TGTAAATAAA
Hmga2	-2316	-2307	0.73705	0.00626	GTTAAAAAAA
Hmga2	-2313	-2304	0.73704	0.00626	AAAAAAAAAA
Hmga2	-2312	-2303	0.73704	0.00626	AAAAAAAAAA
Hmga2	-2311	-2302	0.73704	0.00626	AAAAAAAAAA
Hmga2	-2310	-2301	0.73704	0.00626	AAAAAAAAAA
Hmga2	-2309	-2300	0.73704	0.00626	AAAAAAAAAA
Hmga2	-2308	-2299	0.73704	0.00626	AAAAAAAAAA
Hmga2	-2307	-2298	0.73704	0.00626	AAAAAAAAAA
Hmga2	-2306	-2297	0.73704	0.00626	AAAAAAAAAA
Hmga2	-2305	-2296	0.73704	0.00626	AAAAAAAAAA
Hmga2	-2304	-2295	0.73704	0.00626	AAAAAAAAAA
Hmga2	-2303	-2294	0.73704	0.00626	AAAAAAAAAA

Hmga2	-2302	-2293	0.73704	0.00626	AAAAAAAAAA
Hmga2	-2301	-2292	0.73704	0.00626	AAAAAAAAAA
Hmga2	-2300	-2291	0.73704	0.00626	AAAAAAAAAA
Hmga2	-1517	-1508	0.73704	0.00626	AAAAAAAAAA
Hmga2	-1134	-1125	0.73703	0.00627	TGGAATTAAT
Hmga2	-776	-767	0.73703	0.00627	CACAAATTTT
Hmga2	-1372	-1363	0.73697	0.00631	ATTCAAAATT
Hmga2	-1223	-1214	0.73439	0.00646	TGTGAATTTA
Hmga2	-100	-91	0.73439	0.00646	TGTGAATTTA
Hmga2	311	320	0.73439	0.00646	TGTGAATTTA
Hmga2	401	410	0.73433	0.00652	ACTTAAAATT
Hmga2	290	299	0.7342	0.00661	GTGGATATTT
Hmga2	-2314	-2305	0.73178	0.00665	TAAAAAAAAA
Hmga2	-1370	-1361	0.72916	0.00684	TCAAAATTTA
Hmga2	-1067	-1058	0.72916	0.00684	GGAAAAAAAT
Hmga2	338	347	0.7291	0.00686	TTTTAAATAT
Hmga2	764	773	0.7291	0.00687	AGATATTAAA
Hmga2	-2058	-2049	0.72907	0.00689	AAATAAAATA
Hmga2	-1597	-1588	0.7239	0.00723	ATGAAAAAAC
Hmga2	739	748	0.72376	0.00733	TAGCATTTTC
Hmga2	603	612	0.71854	0.00769	AAACATTTAC
Hmga2	-2246	-2237	0.71607	0.00783	GAGGATTAAG
Hmga2	-2315	-2306	0.71601	0.00784	TTAAAAAAAA
Hmga2	-1369	-1360	0.71595	0.00787	CAAAATTTAT
Hmga2	-1070	-1061	0.7131	0.00817	AAGGGAAAAA
Hmga2	-264	-255	0.71065	0.0083	TGGCATTATC
Hmga2	-57	-48	0.71062	0.00834	CGCTATAAAA
Hmga2	-1520	-1511	0.70813	0.00847	AGCAAAAAAA
Hmga2	-182	-173	0.70809	0.00848	ATTCAATTTC
Hmga2	-1296	-1287	0.70784	0.00865	AAGGGAAAAT
Hmga2	-1174	-1165	0.70559	0.00868	AAGCAATTCA
Hmga2	-1345	-1336	0.70549	0.00871	AAAAAATTTT
Hmga2	-1501	-1492	0.70536	0.00879	ATACATTTTT
Hmga2	-2162	-2153	0.70535	0.0088	AAATATAAAT
Hmga2	-1066	-1057	0.70281	0.00895	GAAAAAAATT
Hmga2	233	242	0.70273	0.00902	GTTGATTTTT
Hmga2	-2255	-2246	0.70033	0.00913	GTGGAATTTG
Jun	638	644	0.81653	0.00012	TGACACA
Jun	-1773	-1767	0.75251	0.00031	TGCCTCA
Jun	601	607	0.71219	0.00037	TGAAACA
Jun	-1485	-1479	0.70509	0.00043	TGAGTCA
Jun	525	531	0.70509	0.00043	TGAGTCA
Meis2	483	490	0.7145	0.00017	ATGACAGA
Meis3	483	490	0.79932	0.00023	ATGACAGA
Meis3	-1832	-1825	0.74258	0.00041	GTGACACC
Meox2	269	278	0.74022	0.00026	GGTAATCATT

Meox2	678	687	0.73638	0.00027	ATTAATTATG
Mzf1	-2111	-2106	1	0	TGGGGA
Mzf1	-203	-198	1	0	TGGGGA
Mzf1	179	184	1	0	TGGGGA
Mzf1	446	451	0.90234	0.00049	GGGGGA
Nkx6-2	-1547	-1540	0.88737	0.00017	TTCATTAA
Nkx6-2	679	686	0.81497	0.00034	TTAATTAT
Nkx6-2	172	179	0.76493	0.00055	ACAATTAT
Pou5f1	-2315	-2301	0.77552	0	TTAAAAAAAAAAAAA
Rxra	-238	-228	0.68566	2.00E-03	ACTGACCTTGG
Snai1	482	491	0.87532	0.00012	CATGACAGAT
Snai1	-1833	-1824	0.75861	0.00054	GGTGACACCT
Sox10	-1428	-1423	1	0	CTTTGT
Sox10	-1476	-1471	0.96781	0.00024	CATTGT
Sox10	798	803	0.96781	0.00024	CATTGT
Sox10	-103	-98	0.79138	0.00049	CTGTGT
Sox10	465	470	0.79138	0.00049	CTGTGT
Sox10	-1249	-1244	0.7592	0.00122	CAGTGT
Sox10	-459	-454	0.7592	0.00122	CAGTGT
Sox10	-1884	-1879	0.73398	0.00146	TTTTGT
Sox10	-1432	-1427	0.73398	0.00146	CTTTCT
Sox10	-1357	-1352	0.73398	0.00146	TTTTGT
Sox10	-298	-293	0.73398	0.00146	TTTTGT
Sox10	513	518	0.73398	0.00146	CTTTCT
Sox10	657	662	0.73398	0.00146	TTTTGT
Sox10	668	673	0.73398	0.00146	TTTTGT
Sox10	-2337	-2332	0.7018	0.00195	TATTGT
Sox10	-1607	-1602	0.7018	0.00195	TATTGT
Sox10	-936	-931	0.7018	0.00195	CATTCT
Sox10	-656	-651	0.7018	0.00195	TATTGT
Sox6	-1608	-1599	0.70823	9.00E-05	TTATTGTTCT
Sp110	-2268	-2260	0.8824	0.00057	CTAAGGGAG
Sp110	-1298	-1290	0.85887	0.00095	GTAAGGGAA
Sp110	-1715	-1707	0.85382	0.00104	AGCAGGGAT
Sp110	-1072	-1064	0.84882	0.00111	ACAAGGGAA
Sp110	-1401	-1393	0.82865	0.00142	TAAAGGGAA
Sp110	-1096	-1088	0.81678	0.00169	TGTAGGGCT
Sp110	-1217	-1209	0.80166	0.002	TTTAGGGGT
Sp110	-1396	-1388	0.79666	0.00209	GGAAGGGCA
Sp110	-51	-43	0.78835	0.00222	AAAAGGGAA
Sp110	-2128	-2120	0.78664	0.00225	ATAAGGGAC
Sp110	442	450	0.7614	0.00268	CAAAGGGGG
Sp110	-2113	-2105	0.72088	0.00348	GTTGGGGAA
Spib	-2220	-2214	1	0	AGAGGAA
Spib	411	417	0.93084	0.00012	TGAGGAA
Spib	-1641	-1635	0.83823	0.00043	TCAGGAA

Spib	615	621	0.83823	0.00043	TCAGGAA
Spib	-2111	-2105	0.77438	0.00055	TGGGGAA
Tbx1	-1010	-1000	0.69139	2.00E-05	ATGTGTGAAGT
Tbx1	-103	-93	0.66422	8.00E-05	CTGTGTGAATT
Tbx3	-1118	-1109	0.79918	0.00016	GATGTGTCAA
Tbx3	-1011	-1002	0.79631	0.00016	GATGTGTGAA
Tead3	-708	-701	0.95172	3.00E-05	ACATTCCT
Zfp202	177	186	0.81687	0.00034	TATGGGGAGA
Zfp202	442	451	0.74741	0.00095	CAAAGGGGGA
Zfp202	-1305	-1296	0.72466	0.00128	ATTTGGGGTA
Zfp354c	-2190	-2185	0.97814	0.00024	GTGGAG
Zfp354c	64	69	0.97814	0.00024	GTGGAG
Zfp354c	149	154	0.88	0.00049	GTGGAC
Zfp354c	290	295	1	0.002	GTGGAT
Zfp637	120	128	0.71756	0.00016	TATTTATGT
Zscan4c	128	136	0.76087	0.00047	TGTGTGTAT
Zscan4c	114	122	0.75467	5.00E-04	TATGTGTAT
Zscan4c	-1883	-1875	0.70051	0.00081	TTTGTGCTT

Table S4. List of primers used for qPCR

Gene	Direction	Sequence 5'-3'
18S	forward	TGCGGCTTAATTTGACT
18S	reverse	ATCAATCTGTCAATCCTGTC
Actin	forward	GATCAAGATCATTGCTCCTCCTG
Actin	reverse	AGGGTGTAAAACGCAGCTCA
B020004J07RIK	forward	TGACAGATGGAAAGATAGGTGCTGC
B020004J07RIK	reverse	ACAAAGATGAGCTGGGAGCACTGG
Cml2	forward	AGAGCCAGAAGTCTCCCCAT
Cml2	reverse	TCATGCCGCTGGAGAACAAT
Dux	forward	CCTCGATCCCTAGCATCTGG
Dux	reverse	CAAAAGCTCTCCCACGAACC
Duxbl1	forward	GAAGCTTTTGAGAAGAATCGG
Duxbl1	reverse	GGGTGGATGAGGAGTTTTCT
Eif1a	forward	TGGGAGACATAGGCAAGAGG
Eif1a	reverse	CTTTGCAGTTCCTGCATTGA
Gapdh	forward	AATGGTGAAGGTCGGTGTG
Gapdh	reverse	GAAGATGGTGATGGGCTCC
Gm12794	forward	TGCTGCCAAATTCCTTTCTC
Gm12794	reverse	GAGAGTTGGCAGCGATTCAT
Gm16367	forward	AGGGGCCTCAAAAGTCAAGT
Gm16367	reverse	CAGCAGGAAAAGGAATCCAA
Gm4340	forward	TGGCTGCCGACTGTACCTTG
Gm4340	reverse	GTCATGACGTCTTTGCTGGA
Nanog	forward	AACCAGTGGTTGAAGACTAGCAATGGTC
Nanog	reverse	TTCCAGATGCGTTCACCAGATAGC
Oct3/4	forward	CCGTGTGAGGTGGAGTCTGGA
Oct3/4	reverse	CGCCGGTTACAGAACCATACTCG
Sp110	forward	GCACAGCACGAAGAACCACAC
Sp110	reverse	GGCCTTACCACAGGTCACAG
Stra8	forward	GAGGCCCAGCATATGTCTAAC
Stra8	reverse	GCTCTGGTTCCTGGTTTAATG
Tcstv1	forward	ATCCTCAGGAACTGAGAACTTCTGG
Tcstv1	reverse	ATCCCATTCGGCAATCCAGC
Tcstv3	forward	TCTCCAGCTGTTGTGGAATAAGTTCAAC
Tcstv3	reverse	CTTCTTGGCTTTATCCATGGATCCCTGAAGGTAAATC
Zscan4	forward	AGTCTGACTGATGAGTGCTTGAAGCC
Zscan4	reverse	GGCCTTGTTGCAGATTGCTGTTG
